# Circulating inflammatory biomarkers, adipokines and breast cancer risk—a case-control study nested within the EPIC cohort

**DOI:** 10.1186/s12916-022-02319-y

**Published:** 2022-04-18

**Authors:** Manon Cairat, Sabina Rinaldi, Anne-Sophie Navionis, Isabelle Romieu, Carine Biessy, Vivian Viallon, Anja Olsen, Anne Tjønneland, Agnès Fournier, Gianluca Severi, Marina Kvaskoff, Renée T. Fortner, Rudolf Kaaks, Krasimira Aleksandrova, Matthias B. Schulze, Giovanna Masala, Rosario Tumino, Sabina Sieri, Chiara Grasso, Amalia Mattiello, Inger T. Gram, Karina Standahl Olsen, Antonio Agudo, Pilar Amiano Etxezarreta, Maria-Jose Sánchez, Carmen Santiuste, Aurelio Barricarte, Evelyn Monninkhof, Anouk E. Hiensch, David Muller, Melissa A. Merritt, Ruth C. Travis, Elisabete Weiderpass, Marc J. Gunter, Laure Dossus

**Affiliations:** 1 Nutrition and Metabolism Branch, International Agency for Research on Cancer, World Health Organization, 150 cours Albert Thomas, 69372 Lyon, CEDEX 08 France; 2 National Institute of Public Health, Centre for Population Health Research, Cuernavaca, Morelos, Mexico City, Mexico; 3 Danish Cancer Society Research Center, Copenhagen, Denmark; 4 Department of Public Health, University of Aarhus, Aarhus, Denmark; 5 Department of Public Health, Faculty of Health and Medical Sciences, University of Copenhagen, Copenhagen, Denmark; 6 Université Paris-Saclay, UVSQ, Inserm, Gustave Roussy, Équipe “Exposome Et Hérédité”, CESP UMR1018, 94805 Villejuif, France; 7 Department of Statistics, Computer Science and Applications (DISIA), University of Florence, Florence, Italy; 8 Division of Cancer Epidemiology, German Cancer Research Center (DFKZ), Heidelberg, Germany; 9 Department Epidemiological Methods and Etiological Research, Leibniz Institute for Prevention Research and Epidemiology-BIPS, Bremen, Germany; 10 Faculty of Human and Health Sciences, University of Bremen, Bremen, Germany; 11 Department of Molecular Epidemiology, German Institute of Human Nutrition Potsdam-Rehbruecke, Nuthetal, Germany; 12 Institute of Nutritional Science, University of Potsdam, Nuthetal, Germany; 13 Cancer Risk Factors and Life-Style Epidemiology Unit, Institute for Cancer Research, Prevention and Clinical Network – ISPRO, Florence, Italy; 14 Hyblean Association for Epidemiological Research AIRE –ONLUS, Ragusa, Italy; 15 Epidemiology and Prevention Unit, Fondazione IRCCS Istituto Nazionale Dei Tumori Di Milano, Via Venezian, Milano, Italy; 16 Cancer Epidemiology Unit, Department of Medical Sciences, University of Turin, Turin, Italy; 17 Dipartimento Di Medicina Clinica E Chirurgia, Federico II University, Naples, Italy; 18 Department of Community Medicine, Faculty of Health Sciences, UiT The Arctic University of Norway, Tromsø, Norway; 19 Unit of Nutrition and Cancer, Catalan Institute of Oncology – ICO, L’Hospitalet de Llobregat, Spain; 20 Nutrition and Cancer Group; Epidemiology, Public Health, Cancer Prevention and Palliative Care Program; Bellvitge Biomedical Research Institute – IDIBELL, L’Hospitalet de Llobregat, Spain; 21 Ministry of Health of the Basque Government, Sub Directorate for Public Health and Addictions of Gipuzkoa, San Sebastian, Spain; 22 Biodonostia Health Research Institute, Epidemiology of Chronic and Communicable Diseases Group, San Sebastián, Spain; 23 Centro De Investigación Biomédica En Red De Epidemiología Y Salud Pública (CIBERESP), Madrid, Spain; 24 Escuela Andaluza De Salud Pública (EASP), Granada, Spain; 25 Instituto De Investigación Biosanitaria Ibs.GRANADA, Granada, Spain; 26 Department of Preventive Medicine and Public Health, University of Granada, Granada, Spain; 27 Department of Epidemiology, Murcia Regional Health Council, IMIB-Arrixaca, Murcia, Spain; 28 Navarra Public Health Institute, Pamplona, Spain; 29 Navarra Institute for Health Research (IdiSNA), Pamplona, Spain; 30 Julius Center for Health Sciences and Primary Care, University Medical Center Utrecht, Utrecht University, Utrecht, The Netherlands; 31 Department Epidemiology and Biostatistics, School of Public Health, Imperial College London, London, UK; 32 University of Hawaii Cancer Center, Cancer Epidemiology Program, 701 Ilalo St., Honolulu, HI 96813 USA; 33 Cancer Epidemiology Unit, Nuffield Department of Population Health, University of Oxford, Oxford, OX3 0NR UK

**Keywords:** Breast cancer, Inflammation, Biomarkers, Menopausal status, Anthropometry

## Abstract

**Background:**

Inflammation has been hypothesized to play a role in the development and progression of breast cancer and might differently impact breast cancer risk among pre and postmenopausal women. We performed a nested case-control study to examine whether pre-diagnostic circulating concentrations of adiponectin, leptin, c-reactive protein (CRP), tumour necrosis factor-α, interferon-γ and 6 interleukins were associated with breast cancer risk, overall and by menopausal status.

**Methods:**

Pre-diagnostic levels of inflammatory biomarkers were measured in plasma from 1558 case-control pairs from the European Prospective Investigation into Cancer and Nutrition (EPIC) cohort. We used conditional logistic regression to estimate the odds ratios (ORs) of breast cancer at blood collection, per one standard deviation increase in biomarker concentration.

**Results:**

Cases were diagnosed at a mean age of 61.4 years on average 8.6 years after blood collection. No statistically significant association was observed between inflammatory markers and breast cancer risk overall. In premenopausal women, borderline significant inverse associations were observed for leptin, leptin-to-adiponectin ratio and CRP [OR= 0.89 (0.77–1.03), OR= 0.88 (0.76–1.01) and OR= 0.87 (0.75–1.01), respectively] while positive associations were observed among postmenopausal women [OR= 1.16 (1.05–1.29), OR= 1.11 (1.01–1.23), OR= 1.10 (0.99–1.22), respectively]. Adjustment for BMI strengthened the estimates in premenopausal women [leptin: OR = 0.83 (0.68–1.00), leptin-to-adiponectin ratio: OR = 0.80 (0.66–0.97), CRP: OR = 0.85 (0.72–1.00)] but attenuated the estimates in postmenopausal women [leptin: OR = 1.09 (0.96–1.24), leptin-to-adiponectin ratio: OR = 1.02 (0.89–1.16), CRP: OR = 1.04 (0.92–1.16)].

**Conclusions:**

Associations between CRP, leptin and leptin-to-adiponectin ratio with breast cancer risk may represent the dual effect of obesity by menopausal status although this deserves further investigation.

**Supplementary Information:**

The online version contains supplementary material available at 10.1186/s12916-022-02319-y.

## Background

Since the discovery of the infiltration of leukocytes into tumour tissue by Rudolf Virchow in 1863, many experimental studies have confirmed the role of the immune system and inflammation in tumour proliferation, angiogenesis and metastasis [1].

The immune system produces tumour-inhibiting cytokines and then activate T-helper cells to destroy transformed cells and enhance anti-tumour response. T-helper cells can be classified in Th1 and Th2: while Th1 are thought to activate antitumour immunity, Th2 downregulates Th1 response and enhance pro-tumoural humoral response. The balance between Th1 and Th2 response is suggested to play a crucial role in breast tumour development and progression [2–4]. Th1 and Th2 subsets are characterized by the production and release of specific patterns of cytokines such as interferon (IFN)-γ, tumour necrosis factor (TNF)-α and interleukin [IL]-1-RA for Th1 and IL-10, IL-6 and IL-13 for Th2.

Chronic inflammation is now recognized as a hallmark of cancer [5] and was demonstrated to promote lymphomas, melanomas and lung cancers [1]. Emerging evidence suggests that inflammation could also be involved in the physiopathology of breast cancer through the production of free radicals and the subsequent DNA damage as well as the promotion of survival of transformed cells [3].

Inflammation is also a well-established characteristic of obesity [6] which is a known risk factor of postmenopausal breast cancer but a protective factor for premenopausal breast cancer [7]. Therefore, inflammation might differently impact breast cancer risk among pre and postmenopausal women and could be an important pathway which links obesity to postmenopausal breast cancer [8].

The most studied inflammatory biomarker within the framework of breast cancer risk is C-reactive protein (CRP), a marker of the acute-phase inflammatory response. In the most recent meta-analysis of twelve prospective studies on CRP-breast cancer associations, a doubling of CRP levels was associated with a 7% higher risk of breast cancer [9]. These results warrant further investigation since most epidemiological studies were not able to rule out reverse causality due to their short follow-up and limited data on breast cancer subtypes or menopausal status. In addition, some studies were not able to consider risk factors such as adiposity or use of exogenous hormones, although they were previously identified as either potential modifiers or confounders of the inflammation-breast cancer association [10–18]. Particularly, we might expect to find an association between inflammatory biomarkers and breast cancer risk only among non-exogenous hormones users, similarly to what is observed for CRP in previous studies [13, 17]. Indeed, exogenous hormone use is a strong risk factor for breast cancer [19, 20] which masks the effect of adiposity on postmenopausal breast cancer risk [21] and may attenuate or obscure the potential effect conferred by inflammation.

With respect to adipokines produced mainly by adipocytes, levels adiponectin or leptin was either not associated [11, 13, 15, 16, 22–25], negatively associated [22, 26] or positively associated [11] with breast cancer risk. Inconsistent results were also found for IL-6 and TNF-α regarding breast cancer risk [13, 15, 22, 27–29]. Finally, epidemiological data on the associations between pre-diagnostic levels of other inflammatory biomarkers and breast cancer risk are limited.

To address these gaps, we conducted a case-control study nested within the European Prospective Investigation into Cancer and Nutrition (EPIC) cohort to evaluate the associations of eleven cytokines and adipokines linked to inflammation or immune function (tumour necrosis factor [TNF]-α, interferon [IFN]-γ, interleukin [IL]-6, IL-8, IL-10, IL-13, IL-17D, IL-1RA, CRP, leptin, adiponectin) with breast cancer risk, overall and by menopausal status.

## Methods

### The EPIC cohort

The EPIC cohort comprises over 153,000 men and 368,000 women aged 35–75 years old and recruited between 1992 and 1998 in 10 Western European countries [9]. At recruitment, dietary, lifestyle, reproductive, medical and anthropometric data were collected through questionnaires [30]. Around 246,000 women from all countries also provided a baseline blood sample. We excluded participants from Greece due to data access issues and from Norway because they did not have information on HER2 status. Blood was collected according to a standardized protocol in France, Germany, Italy, the Netherlands, Spain and the UK [9]. Serum, plasma, erythrocytes and buffy coat aliquots were stored in liquid nitrogen (− 196 °C) in a centralized biobank at International Agency for Research on Cancer (IARC). In Denmark, blood fractions were stored locally in the vapour phase of liquid nitrogen containers (− 150 °C), and in Sweden, they were stored locally at − 80 °C in standard freezers.

All participants provided written informed consent to participate in the EPIC study, which was approved by the ethics committee of the IARC and local ethical committees in EPIC centres.

### Identification of breast cancer incidence

Breast cancer cases were identified during follow-up based on population cancer registries in Denmark, Italy, the Netherlands, Spain, Sweden and the UK, and on a combination of methods, including health insurance records, contacts with cancer and pathology registries and active follow-up of participants and their next of kin in France and Germany.

### Selection of cases and controls

For the current analyses, we excluded the following individuals: men, women with prevalent cancer (excluding non-malignant skin cancer) at blood collection and with no follow-up data. We also excluded participants with no lifestyle information. We then excluded cases if they (i) did not have blood samples available, (ii) had a follow-up time < 2 years, (iii) had a censure date of follow-up later than 31/12/2012 and (iv) did not have information for at least one of the 3 receptor status (ER, PR and HER2). Of note, we excluded cases that occurred within 2 years of blood collection to minimize reverse causality. We end-up with 3035 cases. We randomly selected among them 1560 cases. For each breast cancer case, one control was selected among all female cohort members who were alive and without cancer diagnosis (except non-melanoma skin cancer) at the age of diagnosis of the index case (incident density sampling). Controls were matched to cases on the centre of recruitment, age (± 6 months), menopausal status at blood collection (premenopausal, perimenopausal, postmenopausal, surgically postmenopausal), phase of the menstrual cycle at blood collection for premenopausal women (early follicular; late follicular; periovulatory; midluteal; other luteal), use of exogenous hormone at blood collection, time of the day (± 1 h), and fasting status at blood collection [non-fasting (< 3 h since last meal), in between (3–6 h), fasting (> 6 h), unknown]. Finally, we excluded 2 cases and their matched controls because they were pregnant at blood collection. The final population included 1558 breast cancer cases and 1558 controls (Additional file 1: Fig. S1).

### Inflammatory biomarker assessment

Cytokines (TNF-α, IFN-γ, IL-6, IL-8, IL-10, IL-13, IL-17D, IL-1RA), adipokines (leptin and adiponectin) and CRP were measured on plasma samples in the laboratories of the Nutrition and Metabolism Branch at IARC, by Meso Scale Discovery (a commercially available and highly sensitive immunoassay platform). Samples from cases and matched controls were analysed together in the same analytical batch, and laboratory personnel were blinded as to the case or control status of samples. Three plasma quality control samples were inserted in duplicate within each analytical batch. Mean intra-batch coefficients of variation, calculated on the concentrations from the quality control samples, varied from 2.6% for CRP to 15.8% for IL17-D. Mean inter-batch coefficients of variation varied from 7.8% for IL-8 to 19.1% for IL17-D.

No measurement below the lower limit of quantification (LOQ) was observed for leptin, CRP, IL-8 and IL-1-RA. Measurements below the LOQ represented less than 3% of the measurements for adiponectin, IFN-γ and IL-17-D, less than 8% for IL-6, 22% for IL-10, 25% for TNF-α and around 80% for IL-13 (Additional file 1: Table S1). When biomarker measurements were lower than the LOQ these values were substituted with half the LOQ. Since most of the IL-13 measurements were below the LOQ, IL-13 was dichotomized into values higher or lower than the LOQ.

### Covariates

Information on lifestyle, reproductive/hormonal and medical factors were obtained through baseline questionnaires. Women were considered premenopausal at the time of blood donation when they reported they were still menstruating or had at least 6–9 menses in the past year. Women were considered postmenopausal when they reported fewer than 4 menses in the past year, or when they reported a bilateral ovariectomy. For 3.4% of women with missing or incomplete questionnaire data or who reported a previous hysterectomy or indicated use of exogenous hormones, the menopausal status was determined according to age cut-points. These women were considered as postmenopausal only if they were 55 years old or more and premenopausal when they were less than 42 years of age at blood collection. Women who were not pre or postmenopausal women were classified as perimenopausal/unknown status. All EPIC centres had self-reported information on age at menarche, age at pregnancy, breastfeeding (except Bilthoven and Umeå), number of live births or still births, oral contraceptive and menopausal hormone therapy (MHT) use.

Among participants who provided a blood sample, 95% had data for anthropometric variables measured by a trained health worker. Height, waist and hip circumferences were measured to the nearest centimeter and weight to the nearest kilogram, in light clothing and without shoes. Waist circumference (WC) was measured by either the narrowest torso circumference or midway between the lower ribs and the iliac crest. The hip circumference was measured from either the widest point or over the buttocks. Among the 5% of study subjects with self-reported anthropometric data, 95% were from the Oxford centre. In this centre, self-reported anthropometric data have been validated in a subset of Oxford participants: anthropometric data were also measured by study researchers in order to calibrate self-reported measurements [31]. Body Mass Index (BMI) was constructed by dividing weight by height in meters squared (kg/m^2^). Physical activity levels were estimated using a questionnaire focused on past-year physical activity in occupational, leisure and household domains. A four-level validated physical activity index (Cambridge physical activity index) was derived by combining occupational physical activity together with time participating in cycling and other physical exercises (such as keep fit, aerobics, swimming and jogging) [32]. Participants reported the number of standard glasses of alcohol they consumed daily or weekly during the 12 months before recruitment. These replies were used to estimate the quantity of ethanol consumption per day. Lifetime histories of hypertension and diabetes were self-reported at baseline.

### Statistical analysis

Characteristics of cases and controls were described using mean and standard deviation (SD) or frequency and percentages. Geometric means were used to describe biomarker concentrations among cases and controls. In the following analyses, the biomarkers and the leptin-to-adiponectin ratio were log-transformed. Partial Pearson’s correlations between biomarkers and anthropometric factors, adjusted for age at blood collection and laboratory batch, were estimated among all controls and by menopausal status at blood collection.

We used conditional logistic regression to estimate the odds ratios (ORs) and 95% confidence intervals (CI) of breast cancer, overall and by menopausal status at blood collection, per one SD increase in log-transformed biomarker concentration. All biomarkers were considered as continuous variables, except IL-13 which was dichotomized into values higher and lower than the LOQ. Possible nonlinear effects were modelled using restricted cubic spline models.

Given our study design and the use of conditional logistic regression, all our models are adjusted for the matching variables by construction. We tested as potential confounding factors known breast cancer risk factors (age at first menstrual period, age at first full term pregnancy and parity combined, breastfeeding, ever oral contraceptive use, ever MHT use, level of physical activity, alcohol consumption, education level, height and BMI) and other covariates assessed at recruitment (smoking status, hip circumference, waist-to-hip ratio, hypertension and diabetes) by comparing models of the biomarkers of interest before and after adjustment for each potential confounder. The categories used for these covariates are displayed in Table 1. We also tested to adjust for BMI in category instead of continuous. Only BMI (continuous) or WC (continuous) modified the ORs of overall breast cancer associated with inflammatory biomarkers by more than 0.05 points. Given the correlations between these variables (0.81), those were included separately in two different models. We systematically presented three models. Model 1 was only conditioned on matching factors. Model 2 was further adjusted for BMI. When we adjusted for WC instead of BMI, the results were virtually unchanged (data not shown). Model 3 was further adjusted for known breast cancer risk factors (listed above).

**Table 1:** Selected characteristics of the study population at blood collection (*n* cases/controls =1558/1558)

Variables	Cases	Controls
	Mean (SD) or ***N*** (%)	Mean (SD) or ***N*** (%)
**Breast cancer cases characteristics**		
**Time between blood collection and diagnosis in years**	8.6 (2.8)	–
**Age at diagnosis in years**	61.4 (8.3)	–
**ER positive**		
Negative	305 (19.6)	–
Positive	1253 (80.4)	–
**PR status**		
Negative	509 (31.8)	–
Positive	1104 (68.2)	–
**HER2 status**		
Negative	1224 (78.6)	–
Positive	334 (21.4)	–
**Molecular status**		
ER+PR±HER2+	210 (13.7)	–
ER+PR±HER2−	1043 (68.3)	–
ER−PR−HER2−	160 (10.5)	–
ER−PR−HER2+	115 (7.5)	–
**Sociodemographic, lifestyle and reproductive factors**		
**Age at blood collection in years**	52.5 (7.9)	52.5 (7.9)
**Education level (*N*** **missing=63)**		
Primary/no schooling	557 (37)	571 (37)
Technical/professional/secondary	647 (42)	638 (42)
Longer education	324 (21)	316 (21)
**Time since last meal at blood collection in hours (*N*** **missing=49)**		
< 3 h	706 (46)	712 (46)
≥ 3–≤ 6 h	273 (18)	272 (18)
> 6 h	552 (36)	552 (36)
**Physical activity index (*N*** **missing=27)**		
Inactive	349 (23)	312 (20)
Moderately inactive	573 (37)	610 (40)
Moderately active	346 (22)	335 (22)
Active	282 (18)	282 (18)
**Smoking status (*N*** **missing=25)**		
Never	875 (56)	875 (56)
Former	351 (23)	334 (22)
Smoker	319 (22)	337 (22)
**Alcohol consumption at recruitment in g/day (*N*** **missing=4)**		
Non drinker	223 (14)	220 (14)
> 0–≤ 3	459 (29)	423 (27)
> 3–≤ 12	454 (29)	434 (28)
> 12–≤ 24	256 (16)	270 (17)
> 24	166 (11)	207 (13)
**Age at first menstrual period in years (*N*** **missing=32)**		
≤ 13	977 (63)	961 (62)
**>** 13	568 (37)	578 (38)
Number of full-term pregnancies and age at first full-term pregnancy* (*N* **missing=91)**		
Nulliparous	243 (16)	213 (14)
Age <30 (1-2 children)	714 (47)	739 (47)
Age <30 (≥3 children)	340 (22)	385 (25)
Age ≥30	215 (14)	176 (11)
**Ever breastfed among women with full-term pregnancy**		
No	199 (15)	188 (14)
Yes	1034 (80)	1,069 (80)
Missing	68 (5)	77 (6)
**Use of exogenous hormones at blood collection**	489 (31.4)	489 (31.4)
**Ever use of oral contraceptive at baseline (*N*** **missing=11)**	939 (50.5)	919 (49.5)
**Ever use of menopausal hormone therapy at baseline among postmenopausal women (*N*** **missing=5)**	415 (49.7)	420 (50.3)
**Menopausal status at blood collection (*N*** **missing=0)**		
Premenopausal	413 (26)	413 (26)
Perimenopausal	307 (20)	307 (20)
Postmenopausal	838 (54)	838 (54)
**Anthropometric factors**		
**Weight in kg**		
Among premenopausal women	64.5 (10.7)	64.8 (11.2)
Among perimenopausal women	67.9 (12.3)	66.8 (10.4)
Among postmenopausal women	69.1 (12.6)	66.8 (10.9)
**Height in cm**		
Among premenopausal women	161.9 (6.5)	162.1 (6.6)
Among perimenopausal women	162.6 (6.6)	161.8 (6.8)
Among postmenopausal women	162.1 (6.6)	161.2 (6.5)
**Body mass index in kg/m**^**2**^		
Among premenopausal women	24.7 (4.2)	24.7 (4.1)
Among perimenopausal women	25.7 (4.6)	25.6 (4.0)
Among postmenopausal women	26.3 (4.7)	25.7 (4.1)
**Waist circumference in cm (*N*** **missing=78)**		
Among premenopausal women	77.8 (10.2)	78.0 (10.2)
Among perimenopausal women	81.8 (11.3)	80.3 (11.1)
Among postmenopausal women	83.3 (11.7)	81.5 (10.2)
**Hip circumference in cm (N missing= 78)**		
Among premenopausal women	99.4 (8.2)	99.9 (8.4)
Among perimenopausal women	102.0 (9.1)	101.3 (8.3)
Among postmenopausal women	103.3 (9.5)	101.3 (8.0)
**Waist to hip ratio (*N*** **missing= 78)**		
Among premenopausal women	0.78 (0.06)	0.78 (0.06)
Among perimenopausal women	0.80 (0.07)	0.79 (0.09)
Among postmenopausal women	0.81 (0.07)	0.80 (0.07)
**Comorbidities**		
**History of diabetes (*N*** **missing=60)**	40 (3)	22 (1)
**History of hypertension**		
No	1078 (35)	1094 (35)
Yes	354 (11)	323 (10)
Missing	126 (4)	141 (5)

Abbreviations: *ER*, oestrogen receptor; *HER2*, human epidermal growth factor 2; *PR*, progesterone receptor; *SD*, standard deviation.

Note: Controls were matched to cases on the centre of recruitment, age, menopausal status at blood collection, phase of the menstrual cycle for premenopausal women, use of exogenous hormone at blood collection, time of the day, and fasting status at blood collection

For these variables, missing values were assigned the median (continuous variables) or mode (categorical variables) if they represented less than 5% of the population or were otherwise classified in a “missing” category (breastfeeding and hypertension).

We investigated heterogeneity by menopausal status at blood collection, breast cancer subtypes (ER+PR±HER2+, ER+PR±HER2−, ER−PR−HER2+ and ER−PR−HER2−), age at diagnosis (as a proxy of menopausal status at diagnosis; ≤ 50 years and > 50 years) and time between blood collection and diagnosis (> 2–≤ 5 years, > 5–< 10 years and ≥ 10 years). Chi-square tests/statistics were calculated as the deviations of logistic beta-coefficients observed in each of the subgroups relative to the overall beta-coefficient. We investigated effect modification by WC (≤ 79 cm and > 79 cm), BMI (< 25 kg/m^2^ and ≥ 25 kg/m^2^) and country, by introducing interaction terms in the models using likelihood ratio tests and adjusting for each matching factors.

We performed several sensitivity analyses. First, we restricted our analyses among non-users of exogenous hormones at blood collection. Then, we restricted to postmenopausal women our sub-group analyses by BMI, WC, breast cancer subtypes, age at diagnosis and time between blood collection and diagnosis. Finally, due to a large number of values below the LOQ for IL-10 and TNF-α, we assessed the associations between these two biomarkers and breast cancer risk among women with quantifiable levels of IL-10 and TNF-α.

## Results

### Description of cases and controls

Cases were diagnosed on average 8.6 years after blood collection at a mean age of 61.4 years (Table 1). Most tumours were ER-positive (80.4%), PR-positive (68.2%) and HER2-negative (78.6%). There was no marked difference between participants’ characteristics and mean concentrations of the biomarkers in cases and controls (Table 1 and Additional file 1: Table S1). In postmenopausal women, cases had on average higher anthropometric measures than controls.

### Correlations between inflammatory biomarkers

All biomarkers were moderately correlated with each other, except IL-17D which was not correlated with any other biomarkers (Table 2). The highest correlations were observed between IL-6 and CRP (*r*=0.40), CRP and leptin-to-adiponectin ratio (*r*=0.38), IFN-γ and TNF-α (*r*=0.37), CRP and leptin (*r*=0.34) and IL-10 and TNF-α (*r*=0.33). Similar correlations were observed among pre, peri and postmenopausal women (data not shown).

**Table 2:** Spearman correlation coefficients between pre-diagnostic levels of inflammatory biomarkers and anthropometric factors among controls, adjusted for laboratory batch and age at blood donation

Biomarkers	Adiponectin	Leptin	L to A ratio	CRP	TNF-α	IFN-γ	IL-6	IL-8	IL-10	IL-1-RA	IL-17-D
**Adiponectin**	1										
**Leptin**	**− 0.18**	1									
**L to A ratio**	**− 0.57**	**0.91**	1								
**CRP**	**− 0.22**	**0.34**	**0.38**	1							
**TNF-α**	**−** 0.05	0.08	0.09	**0.16**	1						
**IFN-γ**	**−** 0.02	**−** 0.01	0.01	**0.13**	**0.37**	1					
**IL-6**	**− 0.14**	**0.25**	**0.27**	**0.40**	**0.28**	**0.20**	1				
**IL-8**	0.03	**−** 0.03	**−** 0.04	**−** 0.01	**0.32**	**0.12**	**0.18**	1			
**IL-10**	**−** 0.01	**−** 0.08	**−** 0.06	0.01	**0.33**	**0.27**	0.09	**0.16**	1		
**IL-1-RA**	**− 0.26**	**0.24**	**0.31**	**0.27**	**0.29**	**0.18**	**0.24**	**0.23**	**0.11**	1	
**IL-17-D**	0.11	**−** 0.05	**−** 0.08	**−** 0.07	0.07	**−** 0.01	0.01	0.06	0.07	**−** 0.02	1
**BMI**	**− 0.25**	**0.60**	**0.61**	**0.40**	0.14	0.03	**0.31**	0.02	**−** 0.03	**0.34**	**−** 0.07
**Weight**	**− 0.23**	**0.55**	**0.55**	**0.35**	**0.12**	0.02	**0.27**	**−** 0.01	**−** 0.02	**0.31**	**−** 0.07
**Height**	0.03	**−** 0.09	**−** 0.09	**−** 0.07	**−** 0.02	**−** 0.01	**−** 0.06	**−** 0.05	0.02	**−** 0.03	0.01
**WC**	**− 0.29**	**0.53**	**0.57**	**0.35**	**0.15**	0.05	**0.28**	0.01	**−** 0.01	**0.33**	**−** 0.06
**HC**	**− 0.15**	**0.56**	**0.54**	**0.33**	0.09	0.03	**0.24**	**−** 0.01	**−** 0.01	**0.27**	**−** 0.05
**WHR**	**− 0.27**	**0.24**	**0.32**	**0.20**	**0.14**	0.05	**0.19**	0.03	**−** 0.01	**0.22**	**−** 0.04

*Abbreviations: BMI*, body mass index; *CRP*, c-reactive protein; *HC*, hip circumference; *IFN*, interferon; *IL*, interleukin; *L to A*, leptin to adiponectin; *SD*, standard deviation; *RA*: receptor antagonist; *TNF*, tumour necrosis factor; *WC*, waist circumference; *WHR*, waist-to-hip ratio

Spearman correlation coefficients with *P* values <0.0001 are in bold

### Correlations between inflammatory biomarkers and anthropometric factors

All the anthropometric factors (except height) were moderately or strongly correlated with inflammatory biomarkers. These anthropometric factors showed the highest correlations with leptin-to-adiponectin ratio (*r* between 0.31 and 0.61) and leptin (*r* between 0.24 and 0.60) and were also positively correlated with IL-6, IL-1RA and CRP (*r* between 0.19 and 0.35) while negatively correlated with adiponectin (*r* ≤ − 0.15).

### Associations between inflammatory biomarkers and breast cancer risk, overall and by menopausal status at blood collection

None of the 11 inflammatory biomarkers was significantly associated with the risk of breast cancer in all women combined (Table 3). No heterogeneity by menopausal status was found for adiponectin, TNF-α, IFN-γ, IL-6, IL-8, IL-1RA, IL-17D and IL-13.

**Table 3:** Associations between inflammatory biomarkers and breast cancer risk overall and according to menopausal status at blood collection

		Menopausal status at blood collection				
Biomarkers Models	All women *N* cases/controls=1558/1558 OR^a^ (95% CI)	Premenopausal women *N* cases/controls=413/413 OR^a^ (95% CI)	Perimenopausal women *N* cases/controls=307/307 OR^a^ (95% CI)	Postmenopausal women *N* cases/controls=838/838 OR^a^ (95% CI)	*P*_homogeneity_^c^	*P*_homogeneity_^d^
**Adiponectin**						
Unadjusted	1.03 (0.95–1.12)	1.10 (0.93–1.30)	0.92 (0.74–1.13)	1.04 (0.93–1.16)	0.42	0.59
Adjusted for BMI	1.06 (0.98–1.16)	1.11 (0.93–1.32)	0.92 (0.74–1.15)	1.09 (0.97–1.22)	0.37	0.91
Fully-adjusted^b^	1.06 (0.97–1.16)	1.12 (0.93–1.35)	0.89 (0.70– 1.12)	1.09 (0.97–1.23)	0.25	0.83
**Leptin**						
Unadjusted	1.05 (0.98–1.13)	0.89 (0.77–1.03)	1.00 (0.85 - 1.18)	1.16 (1.05–1.29)	0.01	<0.01
Adjusted for BMI	0.99 (0.90–1.08)	0.83 (0.68–1.00)	0.97 (0.79–1.19)	1.09 (0.96–1.24)	0.06	0.02
Fully-adjusted^b^	0.98 (0.89–1.08)	0.82 (0.67–1.01)	1.02 (0.82–1.27)	1.06 (0.92–1.21)	0.12	0.04
**Leptin to adiponectin**						
Unadjusted	1.03 (0.96–1.11)	0.88 (0.76–1.01)	1.03 (0.87–1.21)	1.11 (1.01–1.23)	0.03	0.01
Adjusted for BMI	0.96 (0.87–1.05)	0.80 (0.66–0.97)	1.01 (0.81–1.25)	1.02 (0.89–1.16)	0.12	0.05
Fully-adjusted^b^	0.95 (0.86–1.05)	0.80 (0.65–0.98)	1.08 (0.86–1.35)	0.99 (0.87–1.13)	0.11	0.08
**CRP**						
Unadjusted	1.01 (0.93–1.09)	0.87 (0.75–1.01)	0.99 (0.84–1.17)	1.10 (0.99–1.22)	0.04	0.01
Adjusted for BMI	0.97 (0.89–1.05)	0.85 (0.72–1.00)	0.97 (0.81–1.17)	1.04 (0.92–1.16)	0.15	0.05
Fully-adjusted^b^	0.97 (0.89–1.06)	0.83 (0.70–0.98)	0.98 (0.80–1.20)	1.04 (0.92–1.17)	0.10	0.03
**TNF-α**						
Unadjusted	1.06 (0.97–1.16)	0.98 (0.82–1.18)	0.98 (0.81–1.18)	1.15 (1.01–1.31)	0.26	0.17
Adjusted for BMI	1.05 (0.95–1.15)	0.98 (0.82–1.18)	0.97 (0.81–1.17)	1.12 (0.98–1.28)	0.36	0.25
Fully-adjusted^b^	1.05 (0.95–1.15)	1.02 (0.84–1.23)	0.90 (0.74–1.11)	1.11 (0.96–1.27)	0.26	0.49
**IFN-γ**						
Unadjusted	0.97 (0.90–1.05)	0.94 (0.83–1.07)	0.89 (0.74–1.06)	1.02 (0.92 - 1.13)	0.35	0.34
Adjusted for BMI	0.97 (0.90–1.05)	0.94 (0.83–1.07)	0.88 (0.73–1.06)	1.02 (0.92–1.13)	0.32	0.34
Fully-adjusted^b^	0.98 (0.91–1.05)	0.94 (0.82–1.08)	0.88 (0.72–1.06)	1.04 (0.93–1.15)	0.26	0.28
**IL-6**						
Unadjusted	1.02 (0.94–1.11)	1.01 (0.87–1.17)	0.96 (0.80–1.16)	1.05 (0.94–1.18)	0.70	0.63
Adjusted for BMI	0.99 (0.91–1.08)	1.01 (0.86–1.16)	0.95 (0.78–1.15)	1.00 (0.89–1.13)	0.87	0.96
Fully-adjusted^b^	0.98 (0.90–1.07)	0.99 (0.84–1.16)	0.93 (0.75–1.14)	1.02 (0.90–1.15)	0.76	0.80
**IL-8**						
Unadjusted	0.96 (0.88–1.06)	1.15 (0.96–1.38)	0.96 (0.78–1.19)	0.89 (0.79–1.01)	0.08	0.02
Adjusted for BMI	0.97 (0.88–1.06)	1.15 (0.96–1.38)	0.96 (0.78–1.19)	0.90 (0.80–1.02)	0.09	0.03
Fully-adjusted^b^	0.96 (0.88–1.06)	1.18 (0.97–1.44)	0.97 (0.77–1.21)	0.90 (0.79–1.02)	0.08	0.02
**IL-10**						
Unadjusted	1.04 (0.96–1.12)	1.12 (0.96–1.30)	0.89 (0.74–1.06)	1.05 (0.95–1.16)	0.14	0.52
Adjusted for BMI	1.04 (0.97–1.12)	1.12 (0.96–1.30)	0.89 (0.74–1.06)	1.06 (0.96–1.18)	0.14	0.60
Fully-adjusted^b^	1.05 (0.97–1.13)	1.20 (1.03–1.41)	0.84 (0.69–1.02)	1.09 (0.98–1.21)	0.02	0.31
**IL-1RA**						
Unadjusted	1.07 (0.99–1.16)	0.97 (0.82–1.14)	1.17 (0.97–1.40)	1.09 (0.98–1.21)	0.31	0.25
Adjusted for BMI	1.04 (0.96–1.14)	0.97 (0.80–1.16)	1.17 (0.97–1.41)	1.03 (0.92–1.15)	0.35	0.58
Fully-adjusted^b^	1.04 (0.96–1.14)	0.94 (0.77–1.14)	1.18 (0.96–1.45)	1.03 (0.92–1.16)	0.29	0.42
**IL-17D**						
Unadjusted	0.93 (0.86–1.01)	0.94 (0.81–1.08)	0.98 (0.83–1.14)	0.90 (0.80–1.02)	0.74	0.70
Adjusted for BMI	0.94 (0.86–1.02)	0.94 (0.81–1.08)	0.98 (0.83–1.15)	0.92 (0.81–1.04)	0.82	0.83
Fully-adjusted^a^	0.94 (0.86–1.02)	0.93 (0.80–1.09)	1.01 (0.86–1.20)	0.92 (0.81–1.05)	0.64	0.88
**IL-13**						
Unadjusted	0.92 (0.76–1.11)	1.22 (0.82–1.81)	0.69 (0.45–1.04)	0.91 (0.71–1.18)	0.14	0.22
Adjusted for BMI	0.93 (0.77–1.12)	1.22 (0.82–1.81)	0.68 (0.45–1.04)	0.93 (0.72–1.21)	0.14	0.26
Fully-adjusted^b^	0.93 (0.77–1.13)	1.20 (0.79–1.83)	0.68 (0.43–1.05)	0.98 (0.75–1.27)	0.17	0.42

Abbreviations: *CI*, confidence interval; *CRP*, c-reactive protein; *IL*, interleukin; *IFN*, interferon; *OR*, odd ratio; *SD*, standard deviation; *RA*: receptor antagonist; *TNF*, tumour necrosis factor

^a^ORs were estimated per 1 SD increase in log-transformed biomarkers concentrations, from logistic regression conditioned on matching variables. For IL-13, ORs were estimated for levels >LOQ compared to <LOQ

^b^ Fully adjusted models included educational level, body mass index, height, physical activity levels, alcohol consumption, age at menarche, age at first full-term pregnancy and parity, ever breastfeed, ever use of contraceptive pills and ever use of menopausal hormonal therapy. Categories used are those displayed in Table 1

^c^Effect modification was calculated by premenopausal, perimenopausal and postmenopausal women

^d^Effect modification was calculated by premenopausal and postmenopausal women

In unadjusted models, statistically significant heterogeneity according to menopausal status was observed for leptin (*P*_homogeneity(pre/peri/post)_=0.01), leptin-to-adiponectin ratio (*P*_homogeneity(pre/peri/post)_=0.03) and CRP (*P*_homogeneity(pre/peri/post)_=0.04, Table 3). Leptin, leptin-to-adiponectin ratio and CRP were inversely associated with breast cancer risk in premenopausal women [OR_1SD_ = 0.89 (0.77–1.03), OR_1SD_ = 0.88 (0.76–1.01) and OR_1SD_ = 0.87 (0.75–1.01), respectively] but positively associated with breast cancer risk in postmenopausal women [OR_1SD_ = 1.16 (1.05–1.29), OR_1SD_ = 1.11 (1.01–1.23), OR_1SD_ = 1.10 (0.99–1.22), respectively]. Adjustment for BMI strengthened the estimates in premenopausal women [leptin: OR_1SD_ = 0.83 (0.68–1.00), leptin-to-adiponectin ratio: OR_1SD_ = 0.80 (0.66–0.97), CRP: OR_1SD_ = 0.85 (0.72–1.00)] but attenuated the estimates in postmenopausal women [leptin: OR_1SD_ = 1.09 (0.96–1.24), leptin-to-adiponectin ratio: OR_1SD_ = 1.02 (0.89–1.16), CRP: OR_1SD_ = 1.04 (0.92–1.16)]. In fully-adjusted models, statistically significant heterogeneity according to menopausal status at blood collection was also observed for IL-10 (P_homogeneity(pre/peri/post)_ = 0.02). Fully adjusted-models showed a significant positive association with breast cancer risk with levels of IL-10 in premenopausal women [OR_1SD_ = 1.20 (1.03–1.41)]. Among perimenopausal women_,_ none of the biomarkers were associated with breast cancer risk neither in crude, BMI-adjusted or fully-adjusted models (Table 3). Similar patterns were found when analyses where stratified by age at diagnosis (Additional file 1: Table S2).

Departure from linearity was indicated for leptin (*P*_non-linearity_ ≤ 0.01), leptin-to-adiponectin ratio (*P*_non-linearity_ = 0.05) and CRP (*P*_non-linearity_ = 0.04). *P*_non-linearity_ for other biomarkers were higher than 0.07. Restricted cubic spline graphical representations for these three biomarkers are presented in Additional file 1 (Figs. S2-S4), overall and by menopausal status. In premenopausal women, the inverse leptin-breast cancer association was linear (*P*_non-linearity_=0.54) while in postmenopausal women there seemed to be a threshold before which no significant association was observed, with a linear positive association observed after that threshold.

### Associations between inflammatory biomarkers and breast cancer risk by breast cancer subtypes and risk factor subgroups

No heterogeneity was found by breast cancer subtypes, except for IL-13 (*P*_homogeneity_ = 0.04; Additional file 1: Table S3) suggesting a significant positive association with breast cancer risk limited to the ER+PR±HER2+ subtype. A significant heterogeneity by the time of follow-up was found for IL-13 (*P*_homogeneity_<0.01; Additional file 1: Table S4) suggesting an increased breast cancer risk for cases diagnosed between two and five years after blood collection [Fully-adjusted models: OR_1SD_ =2.08 (1.18–3.67)], a decreased breast cancer risk for cases diagnosed between five and ten years after blood collection [Fully-adjusted models: OR_1SD_ =0.70 (0.53–0.93)] and no significant association after ten years [Fully-adjusted models: OR_1SD_ =0.93 (0.68–1.29)]. Other inflammatory biomarkers-breast cancer associations were similar according to breast cancer subtypes (*P*_homogeneity_ ≥ 0.14; Additional file 1: Table S3) and follow-up time (*P*_homogeneity_ ≥ 0.09; Supplementary Table S4).

In unadjusted models, statistically significant heterogeneity by BMI was found for leptin and leptin-to-adiponectin ratio (*P*_homogeneity_=0.03 and *P*_homogeneity_=0.03, respectively; Additional file 1: Table S5) suggesting a higher breast cancer risk among women with BMI ≥ 25kg/m^2^ [OR_1SD_ =1.10 (1.01–1.20) and OR_1SD_ =1.08 (0.99–1.18), respectively] but not among women with BMI < 25 kg/m^2^ [OR_1SD_ =0.97 (0.89–1.05) and OR_1SD_ =0.96 (0.88–1.04), respectively]. Adjustment for BMI attenuated the estimates among women with BMI ≥ 25kg/m^2^ [leptin: OR_1SD_ = 1.06 (0.96–1.17) and leptin-to-adiponectin ratio: OR_1SD_ = 1.04 (0.95–1.15)]. There was no heterogeneity by WC (*P*_homogeneity_ ≥ 0.12; Additional file 1: Table S6). As in the main analyses, we did not observe any heterogeneity by breast cancer subtypes, categories of BMI, WC or time between blood collection and diagnosis among postmenopausal women (*P*_homogeneity_ ≥ 0.20). The power was too limited to evaluate the inflammation-breast cancer associations by subgroups among premenopausal women.

### Sensitivity analyses

The associations were virtually unchanged when we restricted the analyses to women who were not exogenous hormone users at the time of blood collection (Additional file 1: Table S7). Significant heterogeneity by country was only observed only for IL-17 (*P*_homogeneity_=0.04; data not shown). Among women with quantifiable levels of IL-10, no association was observed between IL-10 and breast cancer risk, overall and by menopausal status (Additional file 1: Table S8) Among women with quantifiable levels of TNF-α (Additional file 1: Table S9)., a significant heterogeneity by menopausal status was observed for TNF-α with a positive association among premenopausal women [Fully-adjusted models: OR1_SD_ =2.09 (1.11–3.94)], an inverse association among perimenopausal women [Fully-adjusted models: OR1_SD_ = 0.59 (0.33–0.95)] and no association among postmenopausal women [fully-adjusted models: OR_1SD_ = 1.16 (0.89–1.50)].

## Discussion

In this prospective analysis, no association was observed between the circulating inflammatory biomarkers and breast cancer risk when combining pre, peri and postmenopausal women. However, in premenopausal women, leptin, the leptin-to-adiponectin ratio and CRP were inversely associated with breast cancer risk, while in postmenopausal women they were positively associated with breast cancer risk. Taking into account the level of obesity in the analyses attenuated the associations in postmenopausal women but not in premenopausal women.

Our results are not consistent with results from four previous prospective studies suggesting no association between CRP levels and breast cancer risk among premenopausal women [17, 22, 33, 34]. However, similar to our results, three prospective studies reported an inverse association between breast cancer risk with leptin levels [EPIC-Varese cohort: OR_T3 vs. T1_=0.42 (0.21–0.84) [22]; Nurses’ Health Study II cohort: OR _Q4 vs. Q1_ = 0.69 (0.38–1.23) [16]; The Northern Sweden Health and Disease Cohort: OR_T3 vs. T1_= 0.80 (0.52–1.22) [23]]. Our results are also in line with three prospective studies suggesting no association between adiponectin levels and breast cancer risk [Nurses’ Health Study: OR_q4 vs Q1_=1.30 (0.80–1.03) [25]; EPIC-Varese cohort: OR_1SD_ =1.05 (0.79–1.40) [22]; The Northern Sweden Health and Disease Cohort: OR_T3 vs. T1_= 0.56 (0.28–1.11) [23]]. On the contrary, one randomized trial, initially aimed to investigate the effect of low-dose Tamoxifen and Fenretinide on breast cancer risk, found an inverse association between adiponectin levels and breast cancer risk [HRper unit increase = 0.88 (.081–0.96) [26]]. Only the EPIC-Varese study also reported results for IL-6 and TNF-α among premenopausal women and noted a higher breast cancer risk with increasing levels of IL-6 [RR_1SD_ = 1.58 (1.02–2.46)] and TNF-α [RR_1SD_=1.81 (0.91–3.61)] [22]. To our knowledge, no other prospective study has so far investigated the role of other biomarkers on breast cancer development among premenopausal women.

Our results in postmenopausal women, although not statistically significant, are compatible with the latest meta-analysis of twelve prospective studies conducted mainly on postmenopausal women showing that higher levels of CRP were associated with a slight increased breast cancer risk [RR _per doubling of circulating CRP concentration_= 1.07 (1.02–1.12)] [9]. It is important to note that, similar to our study, 10 of the 12 studies included in the meta-analysis observed a non-significant association between CRP levels and breast cancer risk, especially after BMI adjustment [10–12, 18, 24, 27, 28, 34–36]. Prospective studies published since the meta-analysis noted either no association [33] or a positive association between CRP and postmenopausal breast cancer risk [13, 14, 17]. Some also suggested effect modification by body size [10–12, 14] or MHT use [13, 17]. Our results in postmenopausal women are in line with several prospective studies showing that breast cancer risk was not associated with levels of adiponectin [11, 13, 15, 23, 24], IL-6 [13, 22, 27, 28] and TNF-α [13, 22, 28]. However, one case-control study nested within the Malmö Diet and Cancer Cohort found an inverse association between high levels of TNF-α and breast cancer risk [OR_T3 vs T1_=0.65 (0.43–0.99)] [29] while the CLUEII study noted a positive association between TNF-α and breast cancer risk [15]. Several prospective studies have also suggested that breast cancer risk was positively associated with increasing levels of leptin [OR_Q4 vs. Q1_= 1.94 (1.37–2.75)] and the leptin-to-adiponectin ratio [OR_Q4 vs. Q1_= 1.91 (1.36–2.68)] [11] while other reported no association with leptin [13, 15, 22, 23]. Others noted that breast cancer risk was negatively associated with levels of adiponectin [22, 25]. As In ours, some studies observed a strong attenuation of the associations between CRP and leptin and postmenopausal breast cancer risk after adjustment for BMI suggesting that BMI was a confounder in the inflammation-breast cancer association. To our knowledge, no prospective study has so far investigated the role of IFN-γ, IL-13, IL-10, IL-17, Il-8 or IL-1RA in breast cancer development among postmenopausal women.

Inflammation has been shown to contribute carcinogenesis by causing genomic instability, increasing the production of free radicals, inducing epigenetic changes, regulating genes involved in cell cycle, angiogenesis, survival and proliferation [1]. In our study, the measured biomarkers were not associated with overall breast cancer risk. However, our study is the first to observe a dual effect of inflammation on breast cancer risk before and after menopause, similar to what is observed for obesity. Although inflammation is suggested to promote carcinogenesis and leptin is a pro-inflammatory cytokine, our results in premenopausal women are supported by experiments indicating that high levels of leptin might reduce breast cancer through regulation of ovarian folliculogenesis [37] and the reduction of follicular oestradiol secretion [38]. To date, the mechanisms underlying the potential protective role of CRP and leptin-to-adiponectin ratio (a suggested surrogate marker of insulin resistance [39]) in breast carcinogenesis among premenopausal women are unclear and should be explored in experimental studies. Further epidemiological studies should replicate these new findings and explore the potential mediating role of inflammation on the obesity-breast cancer associations. Our study also suggested that the positive associations between inflammation and breast cancer risk in postmenopausal women were confounded by obesity. It is therefore very important to take into account anthropometric factors when evaluating the inflammation-breast cancer associations. Finally, our results are in line with results from previous prospective studies suggesting that the associations between the measured inflammatory biomarkers and breast cancer risk did not differ by breast cancer subtypes [11, 13, 16, 23, 25, 33, 34].

The main strength of our study is the use of a large European population-based prospective cohort which allowed us to select a large number of cases and controls and to prospectively examine associations between inflammatory biomarkers and breast cancer risk according to menopausal status and breast cancer subtypes. In addition, the long follow-up (median of 8.6 years among cases) allowed us to evaluate potential reverse causality due to undiagnosed breast cancer by exploring whether the inflammation-breast cancer associations differed according to time of follow-up. Another advantage was that we had detailed data on anthropometry, lifestyle and reproductive factors and we were able to evaluate whether those factors were confounding or modifying the biomarker-breast cancer associations. However, some results from analysis subgroups were based on relatively small numbers of cases and, because of the number of comparisons evaluated, they might be due to chance. Our study also had a number of limitations. The EPIC cohort is based on volunteers of European ancestry with probably more health conscious behaviours and results cannot be generalized beyond the range of biomarker levels represented by this population. There were a too large number of values below the LOQ for IL-13 and we had to dichotomize this biomarker into values higher or lower than the LOQ. There were also a large number of values below the LOQ for IL-10 and TNF-α and a significant heterogeneity by menopausal status was observed when we assessed the associations between TNF-α and breast cancer risk among women with quantifiable levels of TNF-α. However, it remains difficult to interpret these results due to the high number of values below the LOQ and small sample size. In our study, although we were able to adjust our models for anthropometric measures, it remains very difficult to completely rule out residual confounding of anthropometric measures because of their high degree of correlations with CRP and adipokines. Though, we did not observe any heterogeneity by BMI and WC levels when our models were adjusted for BMI. We were also unable to evaluate the potential mediating role of CRP and leptin in the obesity-breast cancer association due 1) to the lack of statistically significant association between BMI or WC and premenopausal breast cancer in our study population and 2) to the lack of statistically significant association between CRP or leptin levels and postmenopausal breast cancer risk after BMI adjustment. Another limitation is that it is not known to what extent circulating levels of the inflammatory biomarkers are associated with levels in breast tissue and circulating levels of the inflammatory biomarkers may be poor surrogates for local activity. In addition, we were able to capture only a small part of the inflammation process and did not measure chemokines or other ILs specific in driving inflammatory diseases such as IL-5, IL-2 or IL-4. Future studies should examine a more extensive set of markers from different immune pathways that could be relevant to cancer development. Finally, as in many other studies on biomarker-cancer risk, the main limitation of our study is that inflammatory biomarkers were measured only once, even though most of the inflammatory markers have shown good reproducibility over time [40].

## Conclusions

In summary, our study suggests that high levels of CRP, leptin and leptin-to-adiponectin ratio could lower breast cancer risk among premenopausal women but not among postmenopausal women. Whether this simply reflects the dual association of obesity with breast cancer risk by menopausal status requires further investigation in other settings.

## Supplementary Information

**Additional file 1: **Circulating inflammatory biomarkers and breast cancer risk in the EPIC prospective study: Supplementary Tables S1- S7 and Figures S1-S3. **Table S1.** Geometric mean values for inflammatory biomarkers in breast cancer cases and matched controls. **Table S2.** Associations between inflammatory biomarkers and breast cancer risk by age at diagnosis. **Table S3.** Associations between inflammatory biomarkers and breast cancer risk by breast cancer molecular subtypes. **Table S4.** Associations between inflammatory biomarkers and breast cancer risk by time between blood collection and diagnosis. **Table S5.** Associations between inflammatory biomarkers and breast cancer risk by body mass index. **Table S6.** Associations between inflammatory biomarkers and breast cancer risk by waist circumference. **Table S7.** Associations between inflammatory biomarkers and breast cancer risk among non-hormone users at blood collection. **Table S8.** Associations between IL-10 and breast cancer risk overall and according to menopausal status at blood collection, after excluding women with values of IL-10 below the LOQ. **Table S9.** Associations between TNF-α and breast cancer risk overall and according to menopausal status at blood collection, after excluding women with values of TNF-α below the LOQ. **Figure S1.** Flow chart of the study population. **Figure S2.** Association between leptin and breast cancer risk, overall and by menopausal status, allowing for nonlinear effects (restricted cubic spline). **Figure S3.** Association between leptin-to-adiponectin ratio and breast cancer risk, overall and by menopausal status, allowing for nonlinear effects (restricted cubic spline). **Figure S4.** Association between CRP with breast cancer risk, overall and by menopausal status, allowing for nonlinear effects (restricted cubic spline).

## Data Availability

For information on how to submit an application for gaining access to EPIC data and/or biospecimens, please follow the instructions at https://login.research4life.org/tacsgr0epic_iarc_fr/access/index.php.

## Abbreviations

BMI: Body mass index
CI: Confidence interval
EPIC: European Prospective Investigation into Cancer and Nutrition
ER: Estrogen receptor
HER2: Human epidermal growth factor receptor 2
IARC: International Agency for Research on Cancer
IFN: Interferon
IL: Interleukin
LOQ: Limit of quantification
OR: Odds ratio
MHT: Menopausal hormone therapy
PR: Progesterone receptor
SD: Standard deviation
TNF: Tumour necrosis factor
WC: Waist circumference
