# Supplementary material for: Circulating inflammatory biomarkers, adipokines and breast cancer risk—a case-control study nested within the EPIC cohort
Source: BMC Med. 2022 Apr 18;20:118. doi: 10.1186/s12916-022-02319-y (PMC9014562; doi:10.1186/s12916-022-02319-y)
Supplement: Supplementary file 1 — Additional file 1: Circulating inflammatory biomarkers and breast cancer risk in the EPIC prospective study: Supplementary Tables S1- S7 and Figures S1-S3. Table S1. Geometric mean values for inflammatory biomarkers in breast cancer cases and matched controls. Table S2. Associations between inflammatory biomarkers and breast cancer risk by age at diagnosis. Table S3. Associations between inflammatory biomarkers and breast cancer risk by breast cancer molecular subtypes. Table S4. Associations between inflammatory biomarkers and breast cancer risk by time between blood collection and diagnosis. Table S5. Associations between inflammatory biomarkers and breast cancer risk by body mass index. Table S6. Associations between inflammatory biomarkers and breast cancer risk by waist circumference. Table S7. Associations between inflammatory biomarkers and breast cancer risk among non-hormone users at blood collection. Table S8. Associations between IL-10 and breast cancer risk overall and according to menopausal status at blood collection, after excluding women with values of IL-10 below the LOQ. Table S9. Associations between TNF-α and breast cancer risk overall and according to menopausal status at blood collection, after excluding women with values of TNF-α below the LOQ. Figure S1. Flow chart of the study population. Figure S2. Association between leptin and breast cancer risk, overall and by menopausal status, allowing for nonlinear effects (restricted cubic spline). Figure S3. Association between leptin-to-adiponectin ratio and breast cancer risk, overall and by menopausal status, allowing for nonlinear effects (restricted cubic spline). Figure S4. Association between CRP with breast cancer risk, overall and by menopausal status, allowing for nonlinear effects (restricted cubic spline). [file 12916_2022_2319_MOESM1_ESM.docx]

**Additional file 1**

**Circulating inflammatory biomarkers and breast cancer risk in the EPIC prospective study: Supplementary Tables S1- S7 and Figures S1-S3.**

**Supplementary tables.**

Table S1. Geometric mean values for inflammatory biomarkers in breast cancer cases and matched controls.

| **Markers** | **N Missing Values** | **N (%) Values ≤LOQ** | **Geometric Mean**  **(95% CI)** | |
| --- | --- | --- | --- | --- |
|  |  |  | **Cases** | **Controls** |
| **Adiponectin (µg/mL)** | 0 | 7 (0.2) | 10.50 (10.24 – 10.77) | 10.39 (10.13 – 10.64) |
| **Leptin (ng /mL)** | 2 | 0 (0) | 9.05 (8.65 – 9.47) | 8.70 (8.34 – 9.08) |
| **CRP (µg/mL)** | 1 | 0 (0) | 1.12 (1.05 – 1.19) | 1.11 (1.05 – 1.18) |
| **TNF-α (pg/mL)** | 8 | 795 (25.5) | 1.06 (1.03- 1.09) | 1.04 (1.01 – 1.07) |
| **IFN-γ (pg/mL)** | 8 | 2 (0.1) | 2.97 (2.87 – 3.08) | 3.02 (2.92 – 3.13) |
| **IL-6 (pg/mL)** | 8 | 238 (7.6) | 0.43 (0.42 – 0.45) | 0.43 (0.41 – 0.44) |
| **IL-8 (pg/mL)** | 8 | 0 (0) | 2.71 (2.62 – 2.80) | 2.75 (2.65 – 2.86) |
| **IL-10 (pg/mL)** | 8 | 693 (22.2) | 0.13 (0.13 – 0.14) | 0.13 (0.13 – 0.14) |
| **IL-1-RA (pg/mL)** | 9 | 0 (0) | 159.11 (154.87 – 163.45) | 154.51 (150.67 – 158.45) |
| **IL-17-D (pg/mL)** | 9 | 76 (2.4) | 6.67 (6.47 – 6.88) | 6.89 (6.69 – 7.09) |
| **IL-13 (pg/mL)^1^** | 9 | 2,470 (79.3) | - | - |

Abbreviations: CI, Confidence interval; CRP, c-reactive protein; IFN, interferon; IL, interleukin; RA: receptor antagonist; LOQ, limit of quantification; mL, millilitre; pg, picogram; TNF, tumour necrosis factor.

^1^ Geometric means not calculated for IL-13 due to the high proportion of values < LOQ

Table S2. Associations between inflammatory biomarkers and breast cancer risk by age at diagnosis.

| **Biomarkers**  Models |  | **Age at diagnosis ≤ 50 years**  N cases/controls=182/182  OR^1^ (95% CI) | |  | **Age at diagnosis > 50 years**  N cases/controls=1376/1376  OR^1^ (95% CI) | P_homogeneity_ |  |
| --- | --- | --- | --- | --- | --- | --- | --- |
| **Adiponectin** |  | |  | | | | |
| Unadjusted |  | 1.15 (0.91 – 1.45) | |  | 1.02 (0.93 – 1.11) | 0.34 |  |
| Adjusted for BMI |  | 1.13 (0.89 – 1.44) | |  | 1.06 (0.96 – 1.16) | 0.60 |  |
| Fully adjusted^²^ |  | 1.30 (0.98 – 1.75) | |  | 1.05 (0.95 – 1.16) | 0.16 |  |
| **Leptin** |  | |  | | | | |
| Unadjusted |  | 0.85 (0.69 – 1.04) | |  | 1.09 (1.00 – 1.18) | 0.03 |  |
| Adjusted for BMI |  | 0.83 (0.63 – 1.08) | |  | 1.02 (0.92 – 1.13) | 0.15 |  |
| Fully adjusted^²^ |  | 0.86 (0.64 – 1.16) | |  | 1.01 (0.91 – 1.12) | 0.34 |  |
| **Leptin to adiponectin** |  | |  | | | | |
| Unadjusted |  | 0.83 (0.67 – 1.02) | |  | 1.06 (0.98 – 1.15) | 0.03 |  |
| Adjusted for BMI |  | 0.80 (0.61 – 1.04) | |  | 0.98 (0.89 – 1.09) | 0.15 |  |
| Fully adjusted^²^ |  | 0.77 (0.56 – 1.04) | |  | 0.98 (0.88 – 1.08) | 0.14 |  |
| **CRP** |  |  | |  |  |  |  |
| Unadjusted |  | 0.88 (0.71 – 1.08) | |  | 1.03 (0.95 – 1.12) | 0.16 |  |
| Adjusted for BMI |  | 0.89 (0.71 – 1.13) | |  | 0.98 (0.90 – 1.07) | 0.46 |  |
| Fully adjusted^²^ |  | 0.80 (0.60 – 1.05) | |  | 0.98 (0.89 – 1.07) | 0.18 |  |
| **TNF-α** |  | |  | | | | |
| Unadjusted |  | 1.02 (0.78 – 1.32) | |  | 1.07 (0.97 – 1.18) | 0.73 |  |
| Adjusted for BMI |  | 1.03 (0.79 – 1.34) | |  | 1.05 (0.95 – 1.16) | 0.89 |  |
| Fully adjusted^²^ |  | 1.17 (0.84 – 1.61) | |  | 1.04 (0.94 – 1.15) | 0.52 |  |
| **IFN-γ** |  |  | |  |  |  |  |
| Unadjusted |  | 1.06 (0.87 – 1.28) | |  | 0.96 (0.89 – 1.04) | 0.37 |  |
| Adjusted for BMI |  | 1.05 (0.86 – 1.28) | |  | 0.96 (0.88 – 1.04) | 0.38 |  |
| Fully adjusted^²^ |  | 1.12 (0.88 – 1.42) | |  | 0.96 (0.89 – 1.04) | 0.24 |  |
| **IL-6** |  |  | |  |  |  |  |
| Unadjusted |  | 0.92 (0.73 – 1.16) | |  | 1.03 (0.95 – 1.13) | 0.36 |  |
| Adjusted for BMI |  | 0.95 (0.74 – 1.22) | |  | 1.00 (0.92 – 1.09) | 0.69 |  |
| Fully adjusted^²^ |  | 0.87 (0.65 – 1.16) | |  | 1.00 (0.91 – 1.09) | 0.38 |  |
| **IL-8** |  |  | |  |  |  |  |
| Unadjusted |  | 1.24 (0.95 – 1.62) | |  | 0.93 (0.85 – 1.03) | 0.05 |  |
| Adjusted for BMI |  | 1.23 (0.94 – 1.61) | |  | 0.93 (0.85 – 1.03) | 0.06 |  |
| Fully adjusted^²^ |  | 1.20 (0.88 – 1.65) | |  | 0.93 (0.85 – 1.03) | 0.13 |  |
| **IL-10** |  |  | |  |  |  |  |
| Unadjusted |  | 1.26 (1.00 – 1.59) | |  | 1.01 (0.93 – 1.10) | 0.09 |  |
| Adjusted for BMI |  | 1.26 (1.00 – 1.59) | |  | 1.02 (0.94 – 1.10) | 0.09 |  |
| Fully adjusted^²^ |  | 1.33 (1.02 – 1.71) | |  | 1.02 (0.94 – 1.11) | 0.06 |  |
| **IL-1RA** |  |  | |  |  |  |  |
| Unadjusted |  | 0.93 (0.72 – 1.20) | |  | 1.09 (1.00 – 1.19) | 0.25 |  |
| Adjusted for BMI |  | 0.96 (0.73 – 1.25) | |  | 1.05 (0.96 – 1.15) | 0.53 |  |
| Fully adjusted^²^ |  | 0.97 (0.71 – 1.33) | |  | 1.05 (0.96 – 1.16) | 0.62 |  |
| **IL-17D** |  |  | |  |  |  |  |
| Unadjusted |  | 0.90 (0.72 – 1.13) | |  | 0.94 (0.86 – 1.02) | 0.77 |  |
| Adjusted for BMI |  | 0.89 (0.70 – 1.12) | |  | 0.94 (0.86 – 1.03) | 0.66 |  |
| Fully adjusted^²^ |  | 0.88 (0.68 – 1.15) | |  | 0.94 (0.86 – 1.03) | 0.65 |  |
| **IL-13** |  |  | |  |  |  |  |
| Unadjusted |  | 1.32 (0.72 – 2.39) | |  | 0.98 (0.72 – 1.08) | 0.21 |  |
| Adjusted for BMI |  | 1.35 (0.74 – 2.46) | |  | 0.89 (0.73 – 1.09) | 0.20 |  |
| Fully adjusted^²^ |  | 1.12 (0.57 – 2.22) | |  | 0.91 (0.74 – 1.12) | 0.57 |  |

Abbreviations: CI, confidence interval; CRP, c-reactive protein; IL, interleukin; IFN, interferon; RA: receptor antagonist; OR, odd ratio; SD, standard deviation; TNF, tumour Necrosis Factor.

^1^ ORs were estimated per 1 SD increase in log-transformed biomarkers concentrations, from logistic regression conditioned on matching variables. For IL-13, ORs were estimated according to the limit of quantification.

² Fully adjusted models included educational level, body mass index, height, physical activity levels, alcohol consumption, age at menarche, age at first full term pregnancy and parity, ever breastfeed, ever use of contraceptive pills and ever use of menopausal hormonal therapy. Categories used are those displayed in the Table 1.

Table S3. Associations between inflammatory biomarkers and breast cancer risk by breast cancer molecular subtypes.

|  | **Breast cancer molecular subtypes** | | | |  | |
| --- | --- | --- | --- | --- | --- | --- |
| **Biomarkers**  Models | **ER+PR±HER2+**  N cases/controls=62/62  OR^1^ (95% CI) | **ER+PR±HER2-**  N cases/controls=252/252  OR^1^ (95% CI) | **ER-PR-HER2-**  N cases/controls=45/45  OR^1^ (95% CI) | **ER-PR-HER2+**  N cases/controls=32/32  OR^1^ (95% CI) | P_homogeneity_ |  |
| **Adiponectin** |  |  |  |  |  |  |
| Unadjusted | 1.10 (0.89 – 1.35) | 1.04 (0.94 – 1.15) | 1.00 (0.75 – 1.33) | 0.92 (0.67 – 1.26) | 0.82 |  |
| Adjusted for BMI | 1.12 (0.90 – 1.38) | 1.09 (1.00 – 1.21) | 0.96 (0.71 – 1.30) | 0.97 (0.70 – 1.36) | 0.80 |  |
| Fully-adjusted^²^ | 1.14 (0.90 – 1.45) | 1.08 (0.97 – 1.21) | 0.93 (0.66 – 1.31) | 1.08 (0.73 – 1.58) | 0.82 |  |
| **Leptin** |  |  |  |  |  |  |
| Unadjusted | 0.92 (0.76 – 1.11) | 1.10 (1.00 – 1.21) | 0.97 (0.79 – 1.20) | 1.10 (0.85 – 1.43) | 0.30 |  |
| Adjusted for BMI | 0.86 (0.68 – 1.09) | 1.01 (0.90 – 1.14) | 1.07 (0.79 – 1.45) | 1.01 (0.70 – 1.43) | 0.64 |  |
| Fully-adjusted^²^ | 0.91 (0.70 – 1.19) | 1.00 (0.89 – 1.13) | 1.11 (0.79 – 1.54) | 0.99 (0.68 – 1.43) | 0.86 |  |
| **Leptin to adiponectin** | |  |  |  |  |  |
| Unadjusted | 0.90 (0.74 – 1.08) | 1.07 (0.97 – 1.17) | 0.97 (0.78 – 1.21) | 1.11 (0.86 – 1.44) | 0.36 |  |
| Adjusted for BMI | 0.83 (0.65 – 1.05) | 0.96 (0.90 – 1.09) | 1.08 (0.79 – 1.47) | 1.03 (0.70 – 1.45) | 0.54 |  |
| Fully-adjusted^²^ | 0.86 (0.65 – 1.12) | 0.96 (0.85 – 1.08) | 1.12 (0.80 – 1.58) | 0.96 (0.65 – 1.41) | 0.68 |  |
| **CRP** |  |  |  |  |  |  |
| Unadjusted | 0.90 (0.74 – 1.09) | 1.04 (0.95 – 1.14) | 0.86 (0.67 – 1.09) | 1.21 (0.90 – 1.62) | 0.19 |  |
| Adjusted for BMI | 0.88 (0.71 – 1.08) | 0.98 (0.90 – 1.09) | 0.87 (0.67 – 1.14) | 1.17 (0.80 – 1.63) | 0.47 |  |
| Fully-adjusted^²^ | 0.83 (0.66 – 1.05) | 0.99 (0.89 – 1.10) | 0.84 (0.62 – 1.14) | 1.07 (0.71 – 1.61) | 0.45 |  |
| **TNF-α** |  |  |  |  |  |  |
| Unadjusted | 1.01 (0.78 –1.32) | 1.05 (0.95 – 1.18) | 0.84 (0.60 – 1.16) | 1.48 (1.03 – 2.13) | 0.14 |  |
| Adjusted for BMI | 1.01 (0.77 – 1.32) | 1.03 (0.90 – 1.15) | 0.84 (0.60 – 1.18) | 1.46 (1.00 – 2.10) | 0.17 |  |
| Fully-adjusted^²^ | 1.03 (0.77 – 1.39) | 1.04 (0.93 – 1.17) | 0.75 (0.51 – 1.10) | 1.46 (0.92 – 2.32) | 0.18 |  |
| **IFN-γ** |  |  |  |  |  |  |
| Unadjusted | 0.93 (0.77 – 1.13) | 0.99 (0.91 – 1.08) | 0.92 (0.73 – 1.16) | 0.93 (0.68 – 1.28) | 0.88 |  |
| Adjusted for BMI | 0.93 (0.77 – 1.13) | 0.99 (0.90 – 1.08) | 0.92 (0.73 – 1.16) | 0.93 (0.70 – 1.27) | 0.88 |  |
| Fully-adjusted^²^ | 0.96 (0.77 – 1.19) | 1.00 (0.92 – 1.10) | 0.88 (0.68 – 1.14) | 0.77 (0.51 – 1.17) | 0.53 |  |
| **IL-6** |  |  |  |  |  |  |
| Unadjusted | 0.95 (0.75 – 1.19) | 1.06 (0.96 – 1.17) | 0.85 (0.65 – 1.11) | 1.00 (0.75 – 1.34) | 0.40 |  |
| Adjusted for BMI | 0.94 (0.74 – 1.19) | 1.02 (0.90 – 1.13) | 0.87 (0.66 – 1.14) | 0.95 (0.70 – 1.29) | 0.66 |  |
| Fully-adjusted^²^ | 0.90 (0.69 – 1.18) | 1.03 (0.93 – 1.15) | 0.82 (0.59 – 1.12) | 0.67 (0.44 – 1.01) | 0.12 |  |
| **IL-8** |  |  |  |  |  |  |
| Unadjusted | 1.00 (0.79 – 1.26) | 0.99 (0.86 – 1.11) | 0.90 (0.69 – 1.17) | 0.81 (0.59 – 1.09) | 0.58 |  |
| Adjusted for BMI | 1.00 (0.79 – 1.26) | 0.99 (0.90 – 1.11) | 0.89 (0.69 – 1.16) | 0.80 (0.60 – 1.09) | 0.56 |  |
| Fully-adjusted^²^ | 1.01 (0.78 – 1.31) | 1.00 (0.89 – 1.13) | 0.83 (0.61 – 1.12) | 0.72 (0.49 – 1.07) | 0.31 |  |
| **IL-10** |  |  |  |  |  |  |
| Unadjusted | 0.98 (0.79 – 1.22) | 1.02 (0.93 – 1.12) | 0.99 (0.77 – 1.27) | 1.20 (0.92 – 1.56) | 0.68 |  |
| Adjusted for BMI | 0.98 (0.79 – 1.22) | 1.03 (0.93 – 1.12) | 0.97 (0.76 – 1.25) | 1.21 (0.90 – 1.59) | 0.61 |  |
| Fully-adjusted^²^ | 0.98 (0.77 – 1.25) | 1.04 (0.95 – 1.14) | 0.92 (0.69 – 1.22) | 1.21 (0.89 – 1.63) | 0.59 |  |
| **IL-1RA** |  |  |  |  |  |  |
| Unadjusted | 1.06 (0.85 – 1.33) | 1.11 (1.01 – 1.23) | 0.90 (0.70 – 1.15) | 1.02 (0.77 – 1.35) | 0.46 |  |
| Adjusted for BMI | 1.07 (0.83 – 1.37) | 1.05 (1.00 – 1.18) | 0.93 (0.71 – 1.20) | 0.97 (0.70 – 1.30) | 0.76 |  |
| Fully-adjusted^²^ | 1.10 (0.83 – 1.46) | 1.07 (0.96 – 1.19) | 0.90 (0.67– 1.21) | 0.84 (0.60 – 1.18) | 0.41 |  |
| **IL-17D** |  |  |  |  |  |  |
| Unadjusted | 1.02 (0.82 – 1.25) | 0.90 (0.81 – 0.99) | 1.04 (0.80 – 1.36) | 0.96 (0.75 – 1.24) | 0.57 |  |
| Adjusted for BMI | 1.02 (0.82 – 1.25) | 0.90 (0.80 – 1.00) | 1.04 (0.80 – 1.35) | 0.97 (0.80 – 1.25) | 0.63 |  |
| Fully-adjusted^²^ | 1.06 (0.83 – 1.34) | 0.90 (0.81 – 1.00) | 1.04 (0.78 – 1.39) | 0.86 (0.64 – 1.15) | 0.51 |  |
| **IL-13** |  |  |  |  |  |  |
| Unadjusted | 1.90 (1.11 – 3.26) | 0.79 (0.63 – 1.00) | 1.00 (0.56 – 1.78) | 0.93 (0.45 – 1.93) | 0.03 |  |
| Adjusted for BMI | 1.91 (1.11 – 3.28) | 0.79(0.60 – 1.00) | 1.00 (0.56 – 1.79) | 0.98 (0.50 – 2.05) | 0.04 |  |
| Fully-adjusted^²^ | 1.90 (1.06 – 3.40) | 0.92 (0.64 – 1.04) | 0.95 (0.51 – 1.80) | 0.87 (0.38 – 1.97) | 0.07 |  |

Abbreviations: CI, confidence interval; CRP, c-reactive protein; ER, oestrogen receptor; HER2, human epidermal growth factor 2; IL, interleukin; IFN, interferon; OR, odd ratio; PR, progesterone receptor; RA: receptor antagonist; SD, standard deviation; TNF, tumour Necrosis Factor

^1^ ORs were estimated per 1 SD increase in log-transformed biomarkers concentrations, from logistic regression conditioned on matching variables. For IL-13, ORs were estimated according to the limit of quantification.

² Fully adjusted models included educational level, body mass index, height, physical activity levels, alcohol consumption, age at menarche, age at first full term pregnancy and parity, ever breastfeed, ever use of contraceptive pills and ever use of menopausal hormonal therapy. Categories used are those displayed in the Table 1.

Table S4. Associations between inflammatory biomarkers and breast cancer risk by time between blood collection and diagnosis.

|  | **Time between blood collection and diagnosis in years** | | |  | | |
| --- | --- | --- | --- | --- | --- | --- |
| **Biomarkers**  Models | **> 2 - ≤ 5**  N cases/controls=226/226  OR^1^ (95% CI) | **> 5 - < 10**  N cases/controls=797/797  OR^1^ (95% CI) | **≥ 10**  N cases/controls=535/535  OR^1^ (95% CI) | | P_homogeneity_ |  |
| **Adiponectin** |  |  |  |  |  |  |
| Unadjusted | 0.92 (0.72 – 1.16) | 1.06 (0.95 – 1.19) | 1.03 (0.89 – 1.19) | | 0.55 |  |
| Adjusted for BMI | 0.93 (0.73 – 1.19) | 1.11 (0.99 – 1.26) | 1.05 (0.90 – 1.21) | | 0.43 |  |
| Fully-adjusted^²^ | 0.96 (0.73 – 1.26) | 1.12 (0.99 – 1.27) | 1.04 (0.89 – 1.22) | | 0.55 |  |
| **Leptin** |  |  |  |  |  |  |
| Unadjusted | 0.94 (0.78 – 1.13) | 1.09 (0.99 – 1.21) | 1.04 (0.91 – 1.19) | | 0.39 |  |
| Adjusted for BMI | 0.81 (0.63 – 1.04) | 1.02 (0.89 – 1.16) | 1.02 (0.87 – 1.20) | | 0.26 |  |
| Fully-adjusted^²^ | 0.75 (0.57 – 0.99) | 1.01 (0.88 – 1.15) | 1.01 (0.86 – 1.20) | | 0.15 |  |
| **Leptin to adiponectin** | |  |  |  |  |  |
| Unadjusted | 0.98 (0.81 – 1.18) | 1.05 (0.95 – 1.16) | 1.02 (0.90 – 1.17) | | 0.80 |  |
| Adjusted for BMI | 0.87 (0.67 – 1.12) | 0.95 (0.84 – 1.09) | 0.99 (0.84 – 1.17) | | 0.69 |  |
| Fully-adjusted^²^ | 0.79 (0.60 – 1.06) | 0.94 (0.82 – 1.08) | 0.99 (0.83 – 1.17) | | 0.45 |  |
| **CRP** |  |  |  | |  |  |
| Unadjusted | 0.98 (0.81 – 1.18) | 0.98 (0.88 – 1.09) | 1.08 (0.94 – 1.23) | | 0.49 |  |
| Adjusted for BMI | 0.93 (0.75 – 1.15) | 0.91 (0.82 – 1.03) | 1.07 (0.93 – 1.23) | | 0.22 |  |
| Fully-adjusted^²^ | 0.88 (0.70 – 1.11) | 0.89 (0.78 – 1.00) | 1.08 (0.93 – 1.26) | | 0.09 |  |
| **TNF-α** |  |  |  |  |  |  |
| Unadjusted | 1.20 (0.93 – 1.56) | 1.03 (0.91 – 1.17) | 1.05 (0.90 – 1.23) | | 0.60 |  |
| Adjusted for BMI | 1.18 (0.91 – 1.54) | 1.01 (0.89 – 1.15) | 1.05 (0.89 – 1.23) | | 0.59 |  |
| Fully-adjusted^²^ | 1.16 (0.87 – 1.56) | 1.00 (0.88 – 1.14) | 1.06 (0.90 – 1.25) | | 0.63 |  |
| **IFN-γ** |  |  |  | |  |  |
| Unadjusted | 0.96 (0.78 – 1.17) | 0.98 (0.88 – 1.09) | 0.97 (0.86 -1.09) | | 0.97 |  |
| Adjusted for BMI | 0.94 (0.77 – 1.16) | 0.98 (0.89 – 1.09) | 0.97 (0.86 – 1.09) | | 0.94 |  |
| Fully-adjusted^²^ | 0.93 (0.74 – 1.15) | 0.98 (0.88 – 1.10) | 0.97 (0.86 – 1.10) | | 0.89 |  |
| **IL-6** |  |  |  | |  |  |
| Unadjusted | 1.01 (0.82 – 1.24) | 1.05 (0.94 – 1.17) | 0.98 (0.86 – 1.13) | | 0.80 |  |
| Adjusted for BMI | 0.98 (0.79 – 1.22) | 1.01 (0.90 – 1.14) | 0.97 (0.84 – 1.12) | | 0.92 |  |
| Fully-adjusted^²^ | 0.95 (0.75 – 1.21) | 0.99 (0.88 – 1.12) | 0.96 (0.83 – 1.12) | | 0.94 |  |
| **IL-8** |  |  |  | |  |  |
| Unadjusted | 0.94 (0.72 – 1.22) | 1.02 (0.91 – 1.15) | 0.87 (0.74 – 1.03) | | 0.31 |  |
| Adjusted for BMI | 0.94 (0.72 – 1.23) | 1.03 (0.91 – 1.16) | 0.88 (0.74 – 1.03) | | 0.29 |  |
| Fully-adjusted^²^ | 0.97 (0.72 – 1.30) | 1.02 (0.90 – 1.16) | 0.86 (0.72 – 1.03) | | 0.30 |  |
| **IL-10** |  |  |  | |  |  |
| Unadjusted | 1.05 (0.86 – 1.28) | 0.99 (0.89 – 1.09) | 1.12 (0.98 – 1.28) | | 0.31 |  |
| Adjusted for BMI | 1.05 (0.86 – 1.28) | 0.99 (0.89 - 1.10) | 1.13 (0.99 – 1.29) | | 0.32 |  |
| Fully-adjusted^²^ | 1.04 (0.84 – 1.30) | 1.00 (0.90 – 1.12) | 1.13 (0.98 – 1.29) | | 0.43 |  |
| **IL-1RA** |  |  |  | |  |  |
| Unadjusted | 1.22 (1.00 – 1.49) | 1.05 (0.94 – 1.17) | 1.05 (0.91 – 1.21) | | 0.40 |  |
| Adjusted for BMI | 1.22 (0.98 – 1.51) | 0.99 (0.88 – 1.12) | 1.03 (0.89 – 1.20) | | 0.28 |  |
| Fully-adjusted^²^ | 1.18 (0.93 – 1.48) | 0.99 (0.88 – 1.13) | 1.05 (0.90 – 1.22) | | 0.45 |  |
| **IL-17D** |  |  |  | |  |  |
| Unadjusted | 1.00 (0.82 – 1.21) | 0.91 (0.82 – 1.02) | 0.93 (0.80 – 1.09) | | 0.75 |  |
| Adjusted for BMI | 0.99 (0.82 – 1.21) | 0.92 (0.83 – 1.03) | 0.93 (0.80 – 1.09) | | 0.80 |  |
| Fully-adjusted^²^ | 1.00 (0.81 – 1.24) | 0.93 (0.83 – 1.04) | 0.91 (0.77 – 1.07) | | 0.77 |  |
| **IL-13** |  |  |  | |  |  |
| Unadjusted | 2.05 (1.20 – 3.50) | 0.71 (0.54 – 0.94) | 0.95 (0.70 – 1.30) | | <0.01 |  |
| Adjusted for BMI | 2.06 (1.21 – 3.53) | 0.71 (0.54 – 0.94) | 0.96 (0.70 – 1.31) | | <0.01 |  |
| Fully-adjusted^²^ | 2.08 (1.18 – 3.67) | 0.70 (0.53 – 0.93) | 0.93 (0.68 – 1.29) | | <0.01 |  |

Abbreviations: CI, confidence interval; CRP, c-reactive protein; IL, interleukin; IFN, interferon; RA: receptor antagonist; OR, odd ratio; SD, standard deviation; TNF, tumour Necrosis Factor.

^1^ ORs were estimated per 1 SD increase in log-transformed biomarkers concentrations, from logistic regression conditioned on matching variables. For IL-13, ORs were estimated according to the limit of quantification.

² Fully adjusted models included educational level, body mass index, height, physical activity levels, alcohol consumption, age at menarche, age at first full term pregnancy and parity, ever breastfeed, ever use of contraceptive pills and ever use of menopausal hormonal therapy. Categories used are those displayed in the Table 1.

Table S5. Associations between inflammatory biomarkers and breast cancer risk by body mass index.

| **Biomarkers**  Models |  | **BMI < 25 kg/m²**  N cases/controls=792/796  OR^1^ (95% CI) | |  | **BMI ≥ 25 kg/m²**  N cases/controls=766/762  OR^1^ (95% CI) | P_homogeneity_ | |
| --- | --- | --- | --- | --- | --- | --- | --- |
| **Adiponectin** |  | |  | | | |  |
| Unadjusted |  | 1.03 (0.95 – 1.11) | |  | 0.99 (0.92 – 1.08) | 0.48 | |
| Adjusted for BMI |  | 1.03 (0.95 – 1.12) | |  | 1.01 (0.93 – 1.10) | 0.61 | |
| Fully adjusted^²^ |  | 1.03 (0.95 – 1.11) | |  | 1.01 (0.93 – 1.10) | 0.63 | |
| **Leptin** |  | |  | | | |  |
| Unadjusted |  | 0.97 (0.89 – 1.05) | |  | 1.10 (1.01 – 1.20) | 0.03 | |
| Adjusted for BMI |  | 0.94 (0.86 – 1.04) | |  | 1.06 (0.96 – 1.17) | 0.07 | |
| Fully adjusted^²^ |  | 0.95 (0.86 – 1.04) | |  | 1.05 (0.95 – 1.16) | 0.10 | |
| **Leptin to adiponectin** |  | |  | | | |  |
| Unadjusted |  | 0.96 (0.88 – 1.04) | |  | 1.08 (0.99 – 1.18) | 0.03 | |
| Adjusted for BMI |  | 0.94 (0.85 – 1.03) | |  | 1.04 (0.95 – 1.15) | 0.08 | |
| Fully adjusted^²^ |  | 0.94 (0.85 – 1.04) | |  | 1.03 (0.94 – 1.14) | 0.11 | |
| **CRP** |  |  | |  |  |  | |
| Unadjusted |  | 0.99 (0.92 – 1.07) | |  | 1.02 (0.94 – 1.11) | 0.55 | |
| Adjusted for BMI |  | 0.98 (0.91 – 1.06) | |  | 0.99 (0.91 – 1.08) | 0.85 | |
| Fully adjusted^²^ |  | 0.99 (0.91 – 1.07) | |  | 0.98 (0.90 – 1.08) | 0.86 | |
| **TNF-α** |  | |  | | | |  |
| Unadjusted |  | 1.00 (0.92 – 1.09) | |  | 1.04 (0.96 – 1.12) | 0.80 | |
| Adjusted for BMI |  | 1.00 (0.92 – 1.09) | |  | 1.03 (0.95 – 1.11) | 0.93 | |
| Fully adjusted^²^ |  | 1.00 (0.92 – 1.09) | |  | 1.02 (0.95 – 1.11) | 0.98 | |
| **IFN-γ** |  |  | |  |  |  | |
| Unadjusted |  | 0.99 (0.92 – 1.06) | |  | 0.98 (0.91 – 1.06) | 0.83 | |
| Adjusted for BMI |  | 0.99 (0.92 – 1.06) | |  | 0.98 (0.91 – 1.06) | 0.79 | |
| Fully adjusted^²^ |  | 0.99 (0.92 – 1.06) | |  | 0.98 (0.90 – 1.06) | 0.84 | |
| **IL-6** |  |  | |  |  |  | |
| Unadjusted |  | 1.03 (0.95 – 1.11) | |  | 0.99 (0.91 – 1.08) | 0.80 | |
| Adjusted for BMI |  | 1.02 (0.95 – 1.11) | |  | 0.96 (0.88 – 1.05) | 0.57 | |
| Fully adjusted^²^ |  | 1.03 (0.95 – 1.11) | |  | 0.96 (0.88 – 1.05) | 0.50 | |
| **IL-8** |  |  | |  |  |  | |
| Unadjusted |  | 1.03 (0.95 – 1.13) | |  | 0.93 (0.85 – 1.02) | 0.03 | |
| Adjusted for BMI |  | 1.04 (0.95 – 1.13) | |  | 0.93 (0.85 – 1.02) | 0.03 | |
| Fully adjusted^²^ |  | 1.04 (0.95 – 1.13) | |  | 0.93 (0.85 – 1.02) | 0.03 | |
| **IL-10** |  |  | |  |  |  | |
| Unadjusted |  | 1.01 (0.94 – 1.09) | |  | 1.02 (0.95 – 1.10) | 0.85 | |
| Adjusted for BMI |  | 1.01 (0.94 – 1.09) | |  | 1.02 (0.95 – 1.10) | 0.87 | |
| Fully adjusted^²^ |  | 1.01 (0.94 – 1.09) | |  | 1.02 (0.95 – 1.10) | 0.83 | |
| **IL-1RA** |  |  | |  |  |  | |
| Unadjusted |  | 1.01 (0.93 – 1.10) | |  | 1.06 (0.98 – 1.14) | 0.61 | |
| Adjusted for BMI |  | 1.01 (0.92 – 1.10) | |  | 1.03 (0.95 – 1.12) | 0.88 | |
| Fully adjusted^²^ |  | 1.01 (0.92 – 1.10) | |  | 1.03 (0.95 – 1.12) | 0.89 | |
| **IL-17D** |  |  | |  |  |  | |
| Unadjusted |  | 0.94 (0.87 – 1.02) | |  | 0.99 (0.92 – 1.06) | 0.39 | |
| Adjusted for BMI |  | 0.94 (0.87 – 1.02) | |  | 0.99 (0.92 – 1.07) | 0.36 | |
| Fully adjusted^²^ |  | 0.94 (0.87 – 1.02) | |  | 1.00 (0.92 – 1.07) | 0.34 | |
| **IL-13** |  |  | |  |  |  | |
| Unadjusted |  | 1.03 (0.86 – 1.23) | |  | 0.90 (0.74 – 1.09) | 0.19 | |
| Adjusted for BMI |  | 1.03 (0.86 – 1.26) | |  | 0.90 (0.74 – 1.09) | 0.19 | |
| Fully adjusted^²^ |  | 1.02 (0.85 – 1.22) | |  | 0.91 (0.75 – 1.10) | 0.19 | |

Abbreviations: CI, confidence interval; BMI, body mass index; CRP, c-reactive protein; IL, interleukin; IFN, interferon; RA: receptor antagonist; OR, odd ratio; SD, standard deviation; TNF, tumour Necrosis Factor.

^1^ ORs were estimated per 1 SD increase in log-transformed biomarkers concentrations, from logistic regression conditioned on matching variables. For IL-13, ORs were estimated according to the limit of quantification.

² Fully adjusted models included educational level, body mass index, height, physical activity levels, alcohol consumption, age at menarche, age at first full term pregnancy and parity, ever breastfeed, ever use of contraceptive pills and ever use of menopausal hormonal therapy. Categories used are those displayed in the Table 1.

Table S6. Associations between inflammatory biomarkers and breast cancer risk by waist circumference.

| **Biomarkers**  Models |  | **WC ≤ 79 cm**  N cases/controls=748/782  OR^1^ (95% CI) | |  | **WC > 79 cm**  N cases/controls=771/737  OR^1^ (95% CI) | P_homogeneity_ | |
| --- | --- | --- | --- | --- | --- | --- | --- |
| **Adiponectin** |  | |  | | | |  |
| Unadjusted |  | 1.03 (0.95 – 1.13) | |  | 1.00 (0.93 – 1.08) | 0.54 | |
| Adjusted for BMI |  | 1.04 (0.95 – 1.13) | |  | 1.01 (0.94 – 1.10) | 0.61 | |
| Fully adjusted^²^ |  | 1.03 (0.95 – 1.12) | |  | 1.01 (0.94 – 1.09) | 0.63 | |
| **Leptin** |  | |  | | | |  |
| Unadjusted |  | 0.97 (0.89 – 1.06) | |  | 1.06 (0.97 – 1.15) | 0.13 | |
| Adjusted for BMI |  | 0.95 (0.85 – 1.05) | |  | 1.02 (0.93 – 1.13) | 0.21 | |
| Fully adjusted^²^ |  | 0.95 (0.86 – 1.05) | |  | 1.01 (0.92 – 1.12) | 0.33 | |
| **Leptin to adiponectin** |  | |  | | | |  |
| Unadjusted |  | 0.96 (0.88 – 1.05) | |  | 1.05 (0.96 – 1.14) | 0.12 | |
| Adjusted for BMI |  | 0.93 (0.84 – 1.03) | |  | 1.01 (0.91 – 1.11) | 0.21 | |
| Fully adjusted^²^ |  | 0.94 (0.85 – 1.04) | |  | 1.00 (0.91 – 1.11) | 0.30 | |
| **CRP** |  |  | |  |  |  | |
| Unadjusted |  | 0.99 (0.92 – 1.07) | |  | 1.01 (0.93 – 1.10) | 0.65 | |
| Adjusted for BMI |  | 0.99 (0.91 – 1.07) | |  | 0.98 (0.90 – 1.08) | 0.85 | |
| Fully adjusted^²^ |  | 0.99 (0.91 – 1.07) | |  | 0.98 (0.90 – 1.08) | 0.88 | |
| **TNF-α** |  | |  | | | |  |
| Unadjusted |  | 1.01 (0.93 – 1.10) | |  | 1.03 (0.95 – 1.12) | 0.63 | |
| Adjusted for BMI |  | 1.01 (0.93 – 1.10) | |  | 1.02 (0.95 – 1.11) | 0.72 | |
| Fully adjusted^²^ |  | 1.02 (0.94 – 1.11) | |  | 1.02 (0.94 – 1.10) | 0.84 | |
| **IFN-γ** |  |  | |  |  |  | |
| Unadjusted |  | 0.97 (0.90 – 1.04) | |  | 1.00 (0.93 – 1.08) | 0.56 | |
| Adjusted for BMI |  | 0.94 (0.90 – 1.04) | |  | 1.00 (0.93 – 1.08) | 0.58 | |
| Fully adjusted^²^ |  | 0.97 (0.90 – 1.04) | |  | 1.00 (0.92 – 1.07) | 0.58 | |
| **IL-6** |  |  | |  |  |  | |
| Unadjusted |  | 0.99 (0.92 – 1.07) | |  | 1.02 (0.94 – 1.11) | 0.61 | |
| Adjusted for BMI |  | 0.99 (0.92 – 1.07) | |  | 1.00 (0.91 – 1.09) | 0.77 | |
| Fully adjusted^²^ |  | 0.99 (0.92 – 1.08) | |  | 0.99 (0.91 – 1.09) | 0.92 | |
| **IL-8** |  |  | |  |  |  | |
| Unadjusted |  | 1.03 (0.94 – 1.12) | |  | 0.95 (0.87 – 1.05) | 0.27 | |
| Adjusted for BMI |  | 1.03 (0.94 – 1.12) | |  | 0.95 (0.87 – 1.05) | 0.27 | |
| Fully adjusted^²^ |  | 1.03 (0.94 – 1.12) | |  | 0.95 (0.87 – 1.05) | 0.22 | |
| **IL-10** |  |  | |  |  |  | |
| Unadjusted |  | 1.00 (0.93 – 1.08) | |  | 1.03 (0.96 – 1.11) | 0.64 | |
| Adjusted for BMI |  | 1.00 (0.93 – 1.08) | |  | 1.04 (0.96 – 1.11) | 0.65 | |
| Fully adjusted^²^ |  | 1.01 (0.93 – 1.09) | |  | 1.03 (0.96 – 1.11) | 0.69 | |
| **IL-1RA** |  |  | |  |  |  | |
| Unadjusted |  | 1.04 (0.95 – 1.13) | |  | 1.02 (0.95 – 1.11) | 0.95 | |
| Adjusted for BMI |  | 1.04 (0.95 – 1.13) | |  | 1.00 (0.92 – 1.08) | 0.75 | |
| Fully adjusted^²^ |  | 1.00 (0.91 – 1.09) | |  | 1.03 (0.95 – 1.12) | 0.89 | |
| **IL-17D** |  |  | |  |  |  | |
| Unadjusted |  | 0.98 (0.91 – 1.06) | |  | 0.96 (0.89 – 1.03) | 0.78 | |
| Adjusted for BMI |  | 0.98 (0.91 – 1.06) | |  | 0.96 (0.89 – 1.04) | 0.82 | |
| Fully adjusted^²^ |  | 0.98 (0.91 – 1.06) | |  | 0.97 (0.90 – 1.04) | 0.90 | |
| **IL-13** |  |  | |  |  |  | |
| Unadjusted |  | 0.99 (0.82 – 1.20) | |  | 0.92 (0.76 – 1.11) | 0.57 | |
| Adjusted for BMI |  | 0.99 (0.82 – 1.20) | |  | 0.92 (0.77 – 1.11) | 0.60 | |
| Fully adjusted^²^ |  | 0.99 (0.82 – 1.21) | |  | 0.93 (0.77 – 1.12) | 0.54 | |

Abbreviations: CI, confidence interval; CRP, c-reactive protein; IL, interleukin; IFN, interferon; RA: receptor antagonist; OR, odd ratio; SD, standard deviation; TNF, tumour Necrosis Factor; WC, waist circumference.

^1^ ORs were estimated per 1 SD increase in log-transformed biomarkers concentrations, from logistic regression conditioned on matching variables. For IL-13, ORs were estimated according to the limit of quantification.

² Fully adjusted models included educational level, body mass index, height, physical activity levels, alcohol consumption, age at menarche, age at first full term pregnancy and parity, ever breastfeed, ever use of contraceptive pills and ever use of menopausal hormonal therapy. Categories used are those displayed in the Table 1.

Table S7. Associations between inflammatory biomarkers and breast cancer risk among non-hormone users at blood collection.

|  | **Menopausal status at blood collection** | | | |
| --- | --- | --- | --- | --- |
| **Biomarkers**  Models | **Premenopausal women**  N cases/controls=386/386  OR (95% CI) | **Perimenopausal women**  N cases/controls=190/190  OR (95% CI) | **Postmenopausal women**  N cases/controls=493/493  OR (95% CI) | P_homogeneity_ |
| **Adiponectin** |  |  |  |  |
| Unadjusted | 1.12 (0.94 – 1.34) | 0.91 (0.70 – 1.19) | 1.02 (0.88 – 1.17) | 0.42 |
| Adjusted for BMI | 1.12 (0.94 – 1.34) | 0.91 (0.69 – 1.19) | 1.10 (0.95 – 1.28) | 0.40 |
| Fully-adjusted^²^ | 1.15 (0.95 – 1.39) | 0.92 (0.69 – 1.23) | 1.10 (0.94 – 1.29) | 0.45 |
| **Leptin** |  |  |  |  |
| Unadjusted | 0.87 (0.75 – 1.01) | 0.94 (0.78 - 1.14) | 1.31 (1.15 – 1.50) | <0.01 |
| Adjusted for BMI | 0.82 (0.68 – 1.00) | 0.92 (0.72 – 1.17) | 1.19 (1.00 – 1.41) | 0.02 |
| Fully-adjusted^²^ | 0.82 (0.67 – 1.01) | 0.98 (0.75 – 1.27) | 1.20 (1.00 – 1.44) | 0.02 |
| **Leptin to adiponectin** |  |  |  |  |
| Unadjusted | 0.85 (0.73 – 1.00) | 0.97 (0.80 – 1.18) | 1.23 (1.08 – 1.40) | <0.01 |
| Adjusted for BMI | 0.79 (0.65 – 0.96) | 0.96 (0.75 – 1.24) | 1.08 (0.92 – 1.27) | 0.06 |
| Fully-adjusted² | 0.78 (0.63 – 0.96) | 1.01 (0.77 – 1.33) | 1.08 (0.91 – 1.29) | 0.05 |
| **CRP** |  |  |  |  |
| Unadjusted | 0.87 (0.75 – 1.02) | 1.01 (0.83 – 1.24) | 1.09 (0.99 – 1.25) | 0.12 |
| Adjusted for BMI | 0.86 (0.73 – 1.02) | 1.03 (0.82 – 1.28) | 0.97 (0.83 – 1.13) | 0.41 |
| Fully-adjusted^²^ | 0.84 (0.70 – 1.00) | 0.98 (0.76 – 1.26) | 0.95 (0.81 – 1.12) | 0.47 |
| **TNF-α** |  |  |  |  |
| Unadjusted | 0.99 (0.83 - 1.19) | 0.86 (0.68 – 1.08) | 1.20 (1.01 – 1.44) | 0.07 |
| Adjusted for BMI | 0.99 (0.83 - 1.19) | 0.85 (0.67 – 1.09) | 1.14 (0.95 – 1.37) | 0.16 |
| Fully-adjusted^²^ | 1.04 (0.86 – 1.26) | 0.80 (0.61 – 1.04) | 1.16 (0.95 – 1.40) | 0.08 |
| **IFN-γ** |  |  |  |  |
| Unadjusted | 0.91 (0.79 – 1.04) | 0.86 (0.68 – 1.08) | 1.07 (0.93 - 1.23) | 0.14 |
| Adjusted for BMI | 0.91 (0.79 – 1.04) | 0.85 (0.68 – 1.08) | 1.08 (0.94 – 1.24) | 0.11 |
| Fully-adjusted² | 0.91 (0.78 – 1.05) | 0.85 (0.66 – 1.10) | 1.10 (0.95 – 1.27) | 0.10 |
| **IL-6** |  |  |  |  |
| Unadjusted | 1.03 (0.88 – 1.20) | 0.93 (0.75 – 1.16) | 1.15 (0.99 – 1.34) | 0.27 |
| Adjusted for BMI | 1.04 (0.89 – 1.21) | 0.93 (0.74 – 1.17) | 1.05 (0.89 – 1.23) | 0.68 |
| Fully-adjusted^²^ | 1.02 (0.86 – 1.20) | 0.87 (0.67 – 1.14) | 1.01 (0.86 – 1.20) | 0.58 |
| **IL-8** |  |  |  |  |
| Unadjusted | 1.16 (0.97 – 1.40) | 0.93 (0.71 – 1.22) | 0.93 (0.80 – 1.09) | 0.16 |
| Adjusted for BMI | 1.17 (0.97 – 1.41) | 0.93 (0.71 – 1.22) | 0.94 (0.80 – 1.10) | 0.18 |
| Fully-adjusted^²^ | 1.20 (0.98 – 1.46) | 0.98 (0.72 – 1.30) | 0.94 (0.80 - 1.11) | 0.17 |
| **IL-10** |  |  |  |  |
| Unadjusted | 1.12 (0.96 – 1.31) | 0.79 (0.62 – 1.00) | 1.01 (0.89 – 1.15) | 0.05 |
| Adjusted for BMI | 1.12 (0.96 – 1.31) | 0.79 (0.62 – 1.00) | 1.04 (0.91 – 1.18) | 0.05 |
| Fully-adjusted^²^ | 1.19 (1.01 – 1.41) | 0.69 (0.53 – 0.91) | 1.07 (0.93 – 1.23) | <0.01 |
| **IL-1RA** |  |  |  |  |
| Unadjusted | 0.98 (0.82 – 1.16) | 1.11 (0.88 – 1.39) | 1.18 (1.02 – 1.35) | 0.25 |
| Adjusted for BMI | 0.99 (0.82 – 1.19) | 1.13 (0.89 – 1.43) | 1.07 (0.92 – 1.24) | 0.67 |
| Fully-adjusted^²^ | 0.97 (0.80 – 1.18) | 1.10 (0.85 – 1.43) | 1.09 (0.93 – 1.28) | 0.59 |
| **IL-17D** |  |  |  |  |
| Unadjusted | 0.93 (0.80 – 1.09) | 1.02 (0.83 – 1.25) | 0.93 (0.79 – 1.09) | 0.73 |
| Adjusted for BMI | 0.93 (0.80 – 1.09) | 1.02 (0.83 – 1.25) | 0.94 (0.79 – 1.11) | 0.77 |
| Fully-adjusted^1^ | 0.94 (0.80 – 1.10) | 1.05 (0.84 – 1.30) | 0.95 (0.80 – 1.13) | 0.71 |
| **IL-13** |  |  |  |  |
| Unadjusted | 1.09 (0.73 – 1.63) | 0.72 (0.42 – 1.23) | 1.00 (0.72 – 1.38) | 0.46 |
| Adjusted for BMI | 1.09 (0.73 – 1.63) | 0.72 (0.42 – 1.23) | 1.04 (0.75 – 1.44) | 0.43 |
| Fully-adjusted^²^ | 1.07 (0.70 – 1.65) | 0.69 (0.39 – 1.23) | 1.07 (0.76 – 1.51) | 0.41 |

Abbreviations: CI, confidence interval; CRP, c-reactive protein; IL, interleukin; IFN, interferon; RA: receptor antagonist; OR, odd ratio; SD, standard deviation; TNF, tumour Necrosis Factor.

^1^ ORs were estimated per 1 SD increase in log-transformed biomarkers concentrations, from logistic regression conditioned on matching variables. For IL-13, ORs were estimated according to the limit of quantification.

² Fully adjusted models included educational level, body mass index, height, physical activity levels, alcohol consumption, age at menarche, age at first full term pregnancy and parity, ever breastfeed, ever use of contraceptive pills and ever use of menopausal hormonal therapy. Categories used are those displayed in the Table 1.

Table S8. Associations between IL-10 and breast cancer risk overall and according to menopausal status at blood collection, after excluding women with values of IL-10 below the LOQ.

|  |  | **Menopausal status at blood collection** | | | |
| --- | --- | --- | --- | --- | --- |
| Models | **All women**  N cases/controls=1219/1219  OR^1^ (95% CI) | **Premenopausal women**  N cases/controls=332/332  OR^1^ (95% CI) | **Perimenopausal women**  N cases/controls=213/213  OR^1^ (95% CI) | **Postmenopausal women**  N cases/controls=674/674  OR^1^ (95% CI) | P_homogeneity_ |
| Unadjusted | 1.04 (0.92 – 1.17) | 1.14 (0.92 – 1.41) | 0.85 (0.58 – 1.25) | 1.02 (0.88 – 1.19) | 0.42 |
| Adjusted for BMI | 1.04 (0.92 – 1.17) | 1.14 (0.91 – 1.41) | 0.81 (0.55 – 1.20) | 1.03 (0.88 – 1.20) | 0.33 |
| Fully-adjusted^²^ | 1.05 (0.93 – 1.19) | 1.22 (0.96 – 1.54) | 0.72 (0.47 – 1.12) | 1.08 (0.92 – 1.27) | 0.12 |

Abbreviations: CI, confidence interval; IL, interleukin; OR, odd ratio; SD, standard deviation.

^1^ORs were estimated per 1 SD increase in log-transformed biomarkers concentrations, from logistic regression conditioned on matching variables.

² Fully adjusted models included educational level, body mass index, height, physical activity levels, alcohol consumption, age at menarche, age at first full term pregnancy and parity, ever breastfeed, ever use of contraceptive pills and ever use of menopausal hormonal therapy. Categories used are those displayed in the Table 1.

Table S9. Associations between TNF-α and breast cancer risk overall and according to menopausal status at blood collection, after excluding women with values of TNF-α below the LOQ.

|  |  | **Menopausal status at blood collection** | | | |
| --- | --- | --- | --- | --- | --- |
| Models | **All women**  N cases/controls=1167/1667  OR^1^ (95% CI) | **Premenopausal women**  N cases/controls=259/259  OR^1^ (95% CI) | **Perimenopausal women**  N cases/controls=212/212  OR^1^ (95% CI) | **Postmenopausal women**  N cases/controls=696/696  OR^1^ (95% CI) | P_homogeneity_ |
| Unadjusted | 1.08 (0.89 – 1.31) | 1.62 (0.95 – 2.76) | 0.63 (0.40 – 1.01) | 1.18 (0.93 – 1.51) | 0.02 |
| Adjusted for BMI | 1.05 (0.87 – 1.28) | 1.62 (0.95 – 2.76) | 0.63 (0.39 – 1.00) | 1.13 (0.88 – 1.44) | 0.02 |
| Fully-adjusted^²^ | 1.07 (0.88 – 1.31) | 2.09 (1.11 – 3.94) | 0.59 (0.33 – 0.95) | 1.16 (0.89 – 1.50) | 0.01 |

Abbreviations: CI, confidence interval; OR, odd ratio; SD, standard deviation; TNF, tumour Necrosis Factor.

^1^ORs were estimated per 1 SD increase in log-transformed biomarkers concentrations, from logistic regression conditioned on matching variables.

² Fully adjusted models included educational level, body mass index, height, physical activity levels, alcohol consumption, age at menarche, age at first full term pregnancy and parity, ever breastfeed, ever use of contraceptive pills and ever use of menopausal hormonal therapy. Categories used are those displayed in the Table 1.

Figure S1. Flow chart of the study population.

**521 324 EPIC participants**

**13 915 invasive cases**

**Exclusion of women with prevalent cancer at recruitment**

21 212

**Exclusion of women with no lifestyle information**

527

**Exclusion of Greek participants**

15 531

**Exclusion of women with no follow-up**

2701

**Exclusion of males**

153 426

**327 927 EPIC women**

**13 671 invasive cases**

**Exclusion of cases without blood samples**

4048

**Exclusion of cases with a follow-up time < 2 years**

1020

**Exclusion of cases diagnoses after 31/12/2012**

43

**Exclusion of cases with receptors status not available**

5 525

**3035 invasive cases available for sampling.**

Among them, 1560 invasive cases were selected and matched to 1 control

2 cases and controls were excluded because they were pregnant at blood collection. The final population included **1558 cases and 15558 controls.**

Figure S2. Association between leptin and breast cancer risk, overall and by menopausal status, allowing for nonlinear effects (restricted cubic spline).


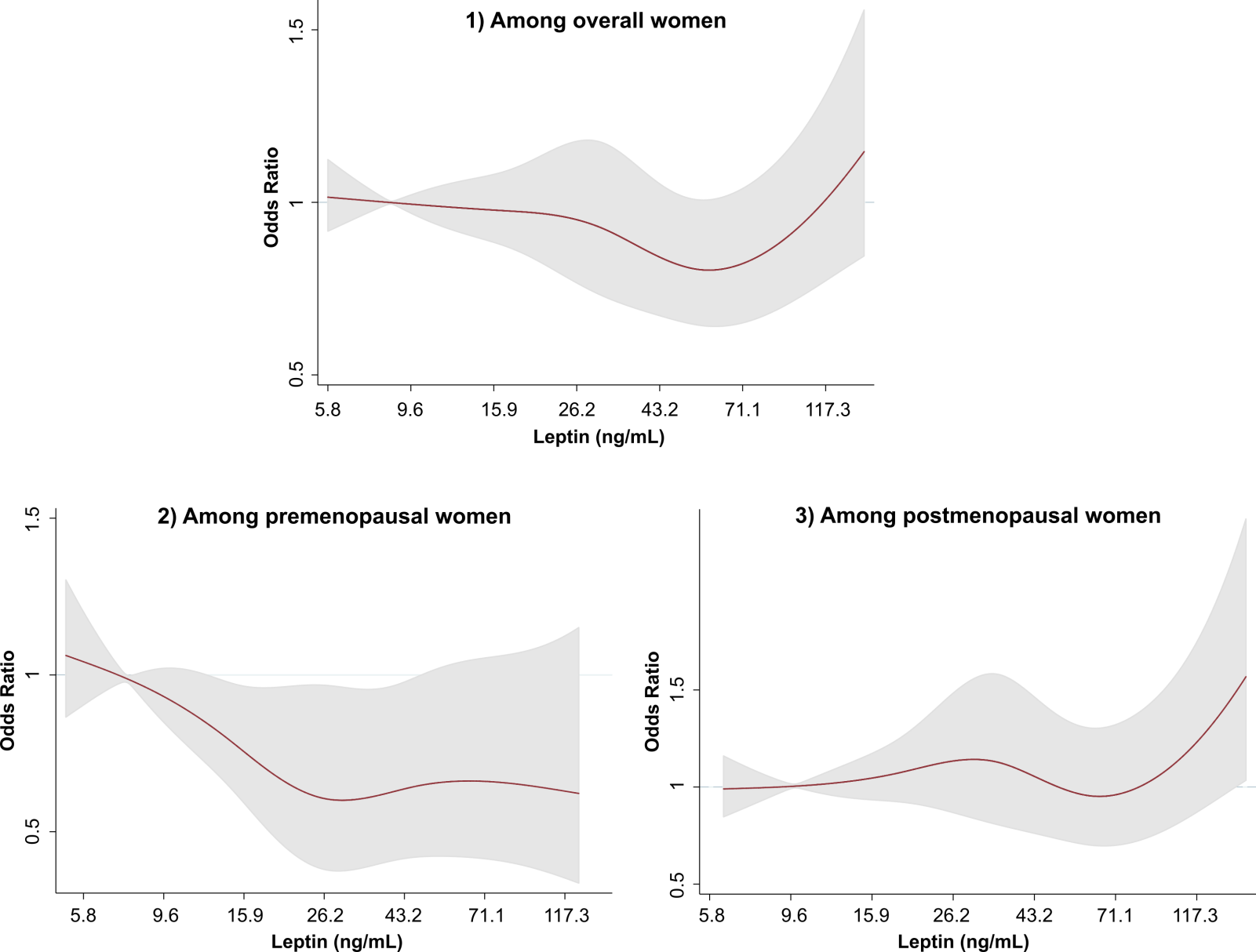


Solid lines indicate the odds ratio, and shaded gray areas indicate the 95% confidence intervals. The references for these restricted cubic spline plots (with five knots placed at the 10th, 25th, 50th, 75th and 95th percentiles) were 8.4 ng/mL among overall women, 7.5 among pre- and 9.6 among postmenopausal women. The models were conditioned on matching factors and adjusted for BMI. The P_non-linearity_ were 0.01 among overall women, 0.54 among premenopausal women and 0.03 among postmenopausal women.

Figure S3. Association between leptin-to-adiponectin ratio and breast cancer risk, overall and by menopausal status, allowing for nonlinear effects (restricted cubic spline).


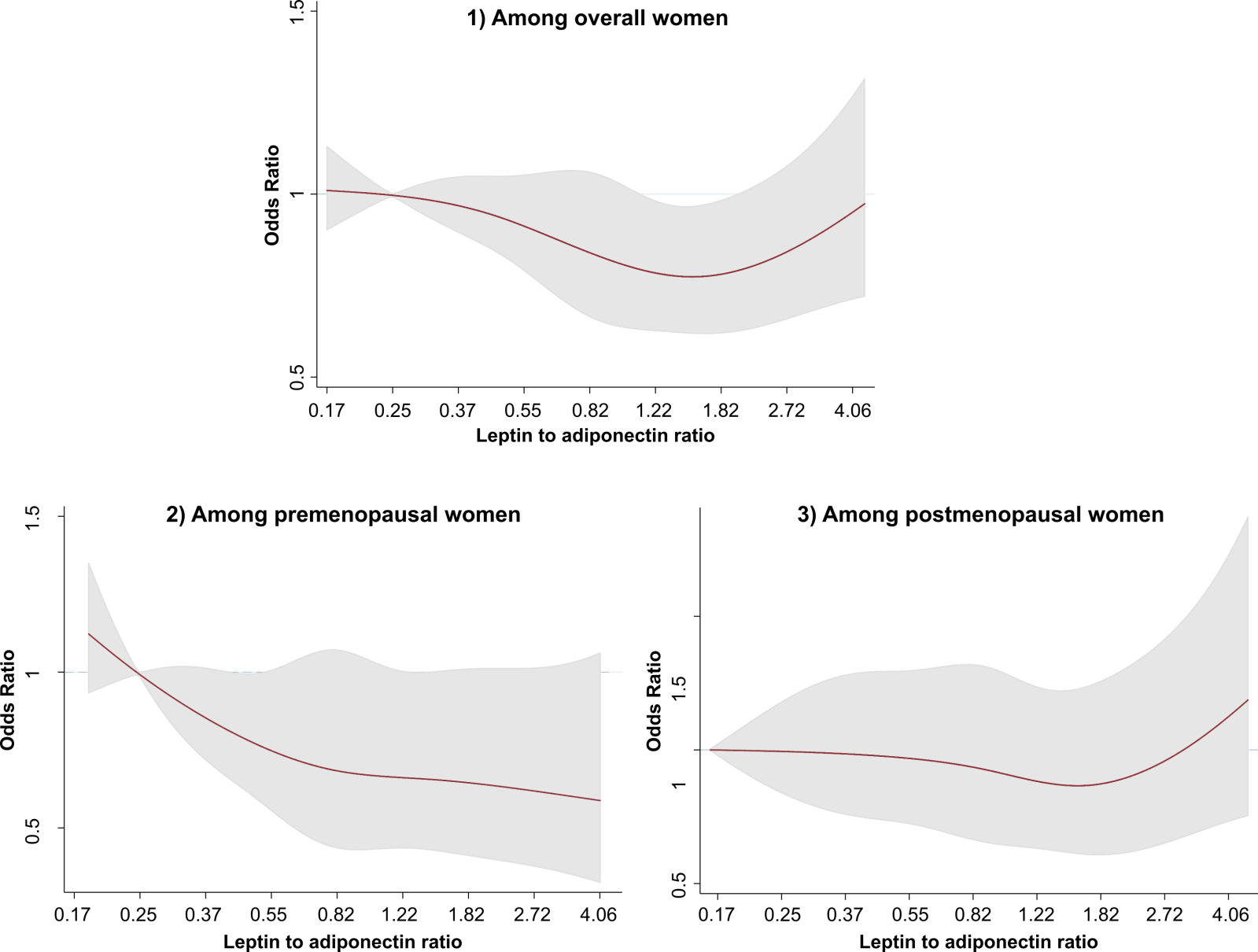


Solid lines indicate the odds ratio, and shaded gray areas indicate the 95% confidence intervals. The references for these restricted cubic spline plots (with five knots placed at the 10th, 25th, 50th, 75th and 95th percentiles) were 0.24 among overall women, 0.24 among pre- and 0.16 among postmenopausal women. The models were conditioned on matching factors and adjusted for BMI. The P_non-linearity_ were 0.05 among overall women, 0.79 among premenopausal women and 0.22 among postmenopausal women.

Figure S4. Association between CRP with breast cancer risk, overall and by menopausal status, allowing for nonlinear effects (restricted cubic spline).


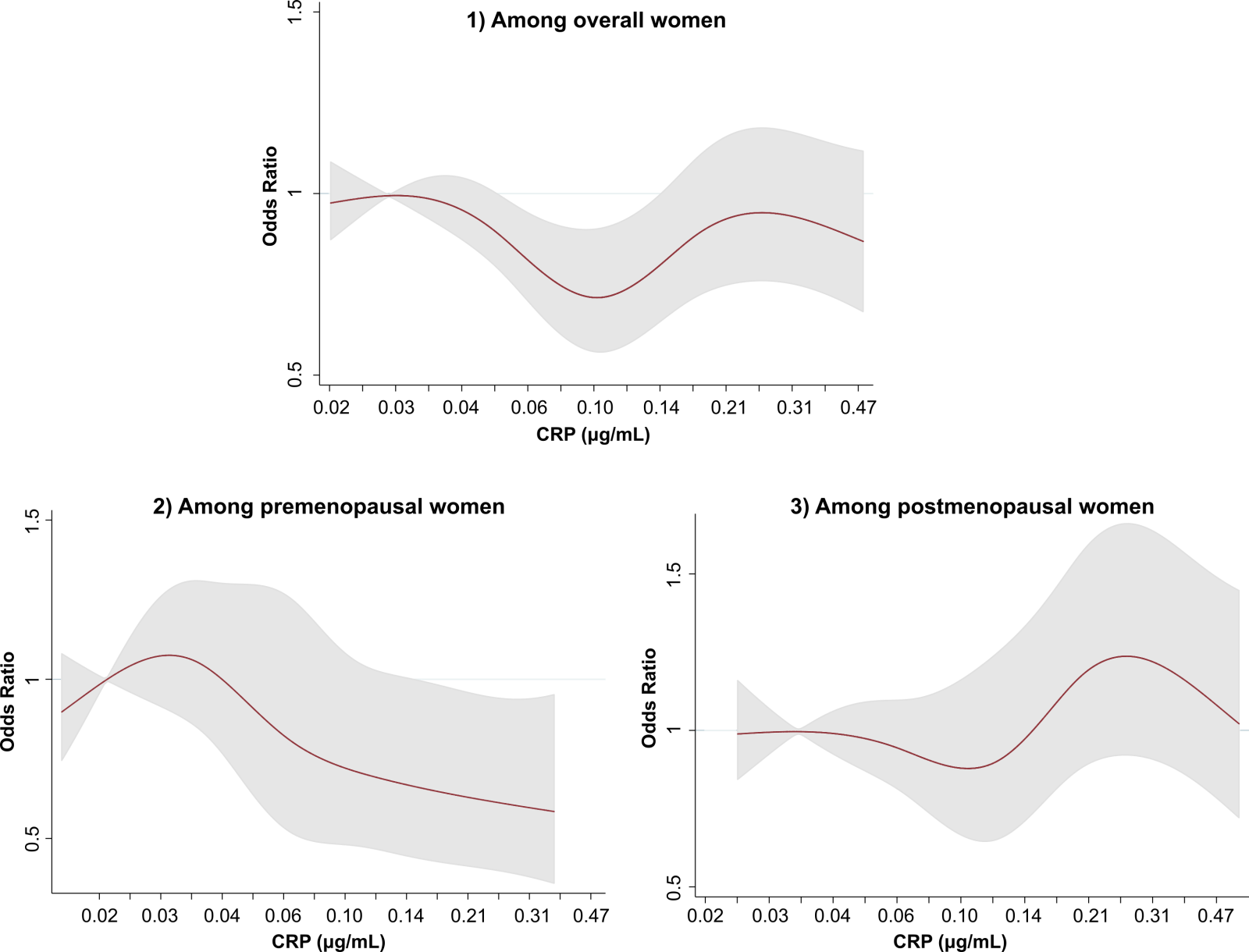


Solid lines indicate the odds ratio, and shaded gray areas indicate the 95% confidence intervals. The references for these restricted cubic spline plots (with five knots placed at the 10th, 25th, 50th, 75th and 95th percentiles) were 0.027 µg/mL among overall women, 0.020 among pre- and 0.034 among postmenopausal women. The models were conditioned on matching factors and adjusted for BMI. The P_non-linearity_ were 0.04 among overall women, 0.37 among premenopausal women and 0.14 among postmenopausal women.
